# Separate To Operate: the Centriole-Free Inner Core of the Centrosome Regulates the Assembly of the Intranuclear Spindle in *Toxoplasma gondii*

**DOI:** 10.1128/mbio.01859-22

**Published:** 2022-09-07

**Authors:** Ramiro Tomasina, Fabiana C. Gonzalez, Érica S. Martins-Duarte, Philippe Bastin, Mathieu Gissot, María E. Francia

**Affiliations:** a Laboratory of Apicomplexan Biology, Institut Pasteur de Montevideo, Montevideo, Uruguay; b Parastiology and Mycology Department, School of Medicine, Universidad de la República, Montevideo, Uruguay; c Departamento de Parasitologia, Universidade Federal de Minas Gerais, Belo Horizonte, Brazil; d Trypanosome Cell Biology Unit, Institut Pasteur, Paris, France; e Lille University, CNRS, Inserm, CHU Lille, Lille, France; f U1019/UMR 9017/CIIL–Center for Infection and Immunity of Lille, Institut Pasteur de Lille, Lille, France; University of Geneva

**Keywords:** endodyogeny, mitosis, mitotic spindle, *Toxoplasma*, ultrastructure expansion microscopy, cell division, centrosome

## Abstract

Centrosomes are the main microtubule-organizing center of the cell. They are normally formed by two centrioles, embedded in a cloud of proteins known as pericentriolar material (PCM). The PCM ascribes centrioles with their microtubule nucleation capacity. Toxoplasma gondii, the causative agent of toxoplasmosis, divides by endodyogeny. Successful cell division is critical for pathogenesis. The centrosome, one of the microtubule organizing centers of the cell, plays central roles in orchestrating the temporal and physical coordination of major organelle segregation and daughter cell formation during endodyogeny. The *Toxoplasma* centrosome is constituted by multiple domains: an outer core, distal from the nucleus; a middle core; and an inner core, proximal to the nucleus. This modular organization has been proposed to underlie T. gondii’s cell division plasticity. However, the role of the inner core remains undeciphered. Here, we focus on understanding the function of the inner core by finely studying the localization and role of its only known molecular marker; TgCep250L1. We show that upon conditional degradation of TgCep250L1 parasites are unable to survive. Mutants exhibit severe nuclear segregation defects. In addition, the rest of the centrosome, defined by the position of the centrioles, disconnects from the nucleus. We explore the structural defects underlying these phenotypes by ultrastructure expansion microscopy. We show that TgCep250L1’s location changes with respect to other markers, and these changes encompass the formation of the mitotic spindle. Moreover, we show that in the absence of TgCep250L1, the microtubule binding protein TgEB1, fails to localize at the mitotic spindle, while unsegregated nuclei accumulate at the residual body. Overall, our data support a model in which the inner core of the T. gondii centrosome critically participates in cell division by directly impacting the formation or stability of the mitotic spindle.

## INTRODUCTION

Centrosomes are the main microtubule-organizing centers (MTOCs) of the cell. In mammalian cells, the centrosome is formed by two microtubule-based barrels, known as centrioles, which display a highly conserved, 9-fold radial symmetry of triplet microtubules. Centrioles reside within a complex matrix of proteins, collectively known as the pericentriolar material (PCM). The PCM ascribes centrioles with their microtubule nucleation capacity. The centrosome’s microtubule organization capacity plays pivotal roles in cellular life, impacting cell shape and polarity, organizing the formation of motile structures, and participating in karyokinesis.

The phylum *Apicomplexa* is a large group of protozoan parasites, consisting of more than 6,000 species (1). Apicomplexans cause important human and animal diseases, including toxoplasmosis, malaria, neosporosis, and cryptosporidiosis. Toxoplasmosis is caused by Toxoplasma gondii, arguably the most successful parasitic organism of warm-blooded animals in the world. It is estimated that around 30% of the human population is infected with this parasite. The most severe outcomes by T. gondii infection are due to reactivation of chronic infections, primo-infections in immunocompromised individuals, and congenital transmission (2, 3). The latter could lead to miscarriage or irreversible sequelae in the newborn.

T. gondii actively invades virtually any nucleated cell. Once inside the cell, it replicates, scaling its numbers rapidly and exponentially, eventually causing host cell lysis. Newly released parasites can subsequently invade healthy neighboring cells perpetuating the infection and exponentially upscaling the damage. The fast dividing form of the parasites, known as the tachyzoite, follows a cell division scheme known as endodyogeny. Endodyogeny consists of a semiclosed nuclear mitosis—i.e., no appreciable chromatin condensation or nuclear envelope breakdown occurs—concomitant with the assembly of two daughter cells within the mother cell (4).

Tachyzoites bear two MTOCs: the apical polar ring (APR) and the centrosome. The APR is involved in nucleating the cortical microtubules that shape and permit parasite motility (5). The centrosome in T. gondii has been shown to orchestrate the temporal and spatial coordination of nuclear mitosis and daughter cell formation (6, 7). On one hand, the centrosome nucleates the mitotic spindle microtubules, impacting chromatin organization and nuclear segregation, while on the other hand, the centrosome organizes the seeds of new cells by physically positioning the offspring’s APR, thereby spatially and temporally linking new daughter cell formation with nuclear content segregation (8).

Recently, the centrosome in T. gondii was shown to be constituted by three distinct protein localization domains. Initially, an outer core, distal from the nucleus, and an inner core, proximal to the nucleus, were identified (9). These domains were described based on the localization of centrosomal protein homologs. A homolog of Centrin1, an EF-hand calcium binding protein and a *bona fide* marker of centrioles in many species, was shown to localize at the outer core. Likewise, SAS6, a protein involved in forming the structure responsible for ascribing centrioles with their characteristic geometry, known as the cartwheel, colocalizes with TgCentrin1. SfiI and γ-tubulin orthologs are also found at the outer core (9, 10). These observations led to the proposal that centrioles likely reside within the outer domain (9). However, this has not been experimentally validated. On the other hand, TgCep250L1 (TgME49_290620), a distant homolog of CEP250, a centrosomal protein involved in centriole cohesion, localizes to the inner core exclusively (9, 11). An additional CEP250 homolog, TgCep250 (TgME49_212880) localizes to both the inner and outer cores (9, 11). Experimental manipulation of TgCep250 causes physical separation of the cores and the concomitant dysregulation of cytosolic and nuclear events during cell division. This protein has been proposed to bridge cohesion between the two opposite cores (11). More recently, a third protein localization domain, in between the initially described outer and inner cores, was identified. The “middle” core houses TgCep530 (TgME49_246190); a mutant of this protein loses synchrony between cytokinesis and karyokinesis and exhibits outer core fragmentation, indicating that this domain is also important for centrosomal homeostasis (12).

Although the outer core has been proposed to regulate aspects of daughter cell formation and the middle core has been shown to play a role in cohesion and outer core stability, the role played by the inner core remains experimentally unexplored. To gain further insight into this domains, we focus here on the characterization of TgCep250L1’s function, as a proxy to the role of the centrosomal inner core in cell division in T. gondii.

## RESULTS

### TgCep250L1 is required for parasite growth and survival.

**FIG 1:**
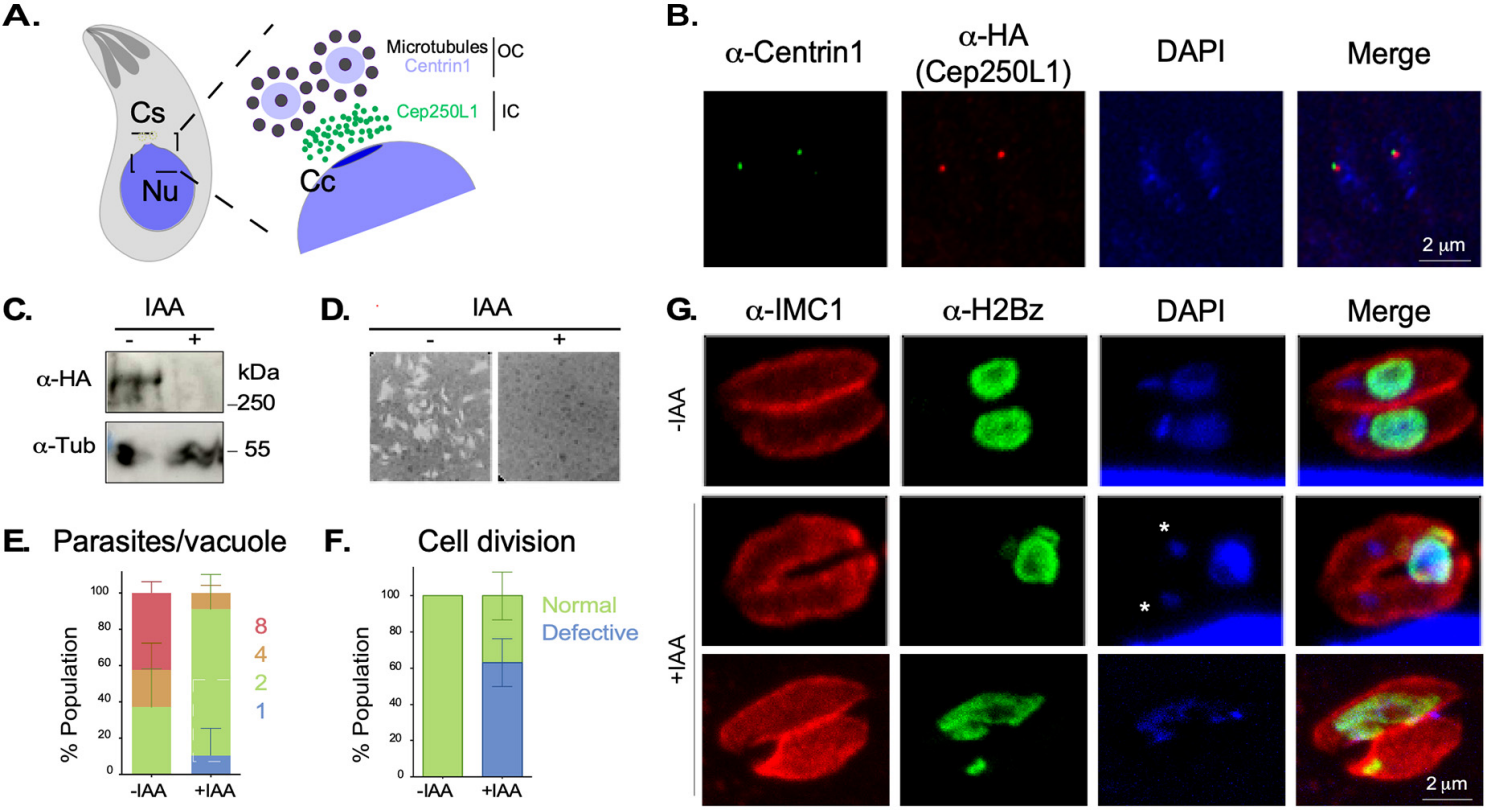
Conditional knockdown of TgCep250L1 causes nuclear segregation defects. (A) Schematic representation of the bipartite centrosome of T. gondii. The centrosome (Cs) of T. gondii is organized into two core domains. The outer core houses proteins such as Centrin1 and has been proposed to house two parallel centrioles displaying a 9-fold symmetry of single microtubules. The relative position of TgCep250L1, previously approximated by structured illumination super-resolution microscopy, is shown. The centrocone (Cc) is a nuclear (Nu) envelope elaboration apposed to the centrosome present throughout the cell cycle. (B) The fusion protein TgCep250L1-mAID-3HA correctly localizes to the centrosome. The results of an IFA of TgCep250L1-mAID-HA parasites stained with anti-Centrin1 antibody (green), anti-HA antibody for TgCep250L1-mAID-3HA (red), and DAPI (blue) are shown. A maximum intensity projected z-stack spanning the entire parasite is shown. (C) TgCep250L1-mAID-3HA rapidly degrades upon addition of IAA to the growth media. Western blot analysis of total protein extracts from the TgCep250L1-mAID-HA strain treated or not with IAA for 24 h. (D) TgCep250L1 is required for tachyzoite proliferation. HFF monolayers were infected with equal numbers of TgCep250L1-mAID-HA and treated or not, as indicated, for a week. Note that while untreated parasites (–IAA) are able to generate lysis plaques in the monolayer, treated parasites (+IAA) do not proliferate. (E) TgCep250L1 knockdown stalls parasite replication. The numbers of TgCep250L1-mAID-HA parasites per vacuole, treated as indicated, were quantified. The data shown are the averages of three independent experiments. A minimum of 50 vacuoles were quantified per replicate. The data plotted are the averages of three independent experiments. Error bars represent the standard deviations of the mean. (F) TgCep250L1 knockdown yields parasites displaying aberrant cell division phenotypes. The numbers of parasites displaying mis-segregated or no nuclei were quantified by IFA. The data shown are the averages of three independent experiments A minimum of 50 vacuoles were quantified per replicate. The data plotted are the averages of three independent experiments. Error bars represent the standard deviations of the mean. (G) TgCep250L1 depletion causes nuclear mis-segregation. TgCep250L1-mAID-HA parasites, treated as indicated for 24 h, were stained with anti-IMC1 (red; pellicle marker), anti-H2Bz (green; nucleus), and DAPI (blue; DNA marker). Am asterisk marks the apicoplast DNA labeled with DAPI.

To assess the role of the centrosomal inner core of T. gondii, we generated a knockdown strain of its only identified marker, TgCep250L1, by inserting a mini-auxin inducible degron sequence (mAID) (13), followed by a triple-hemagglutinin epitope tag (3HA), in frame with the TgCep250L1’s coding sequence in a Tir1-expressing parental cell line (Fig. 1A; see also Fig. S1A in the supplemental material). Successful generation of TgCep250L1-mAID-3HA was corroborated by PCR (see Fig. S1B). Immunofluorescence assays (IFAs) with anti-HA antibodies to visualize TgCep250L1 and anti-Centrin as a proxy for the centrosome position show that the TgCep250L1-mAID-HA fusion correctly localizes to the organelle (Fig. 1B).

10.1128/mbio.01859-22.1 FIG S1 Strategy used for obtaining TgCEP250L1-mAID-3HA strain. (A) TgCEP250L1iKD was obtained by replacing its 3′ end with the mAID degron sequence in a Tir1-expressing parental strain, using CRISPR/Cas9 directed to the desired locus. (B) PCR showing the correct insertion of mAID-3HA in the locus of TgCEP250L1. The expected band of ~2 kb in the parasites with the insertion is observed. (C) Western blot showing the correct expression of the protein TgCEP250L1-mAID-3HA and its inducible knockdown after 0.5, 2, 4, and 6 h of incubation with IAA. (D) IFA of TgCEP250L1-mAID-3HA showing the depletion of the protein at 0.5, 2, 4, and 6 h after incubation with IAA. Download FIG S1, PDF file, 0.1 MB. Copyright © 2022 Tomasina et al. 2022 Tomasina et al. https://creativecommons.org/licenses/by/4.0/ This content is distributed under the terms of the Creative Commons Attribution 4.0 International license.

Degradation of TgCep250L1-mAID-HA is rapidly triggered by the exogenous addition of the auxin indole acetic acid (IAA) to the growth media. Protein knockdown is observed by Western blotting (WB) as early as 30 min after IAA addition (see Fig. S1C and Fig. S2A and B), becoming undetectable in 2 h both by WB and IFA (Fig. 1C; see also Fig. S1C).

10.1128/mbio.01859-22.2 FIG S2 TgCEP250L1 is completely depleted upon the incubation with IAA. (A) Uncropped Western blot image of the cropped image shown in Fig. S1C. (B) Uncropped Western blot image of the cropped image shown in Fig. 1C. Download FIG S2, PDF file, 0.09 MB. Copyright © 2022 Tomasina et al. 2022 Tomasina et al. https://creativecommons.org/licenses/by/4.0/ This content is distributed under the terms of the Creative Commons Attribution 4.0 International license.

To assess the effect of TgCep250L1 knockdown on parasite survival, we performed plaque assays. Protein knockdown was triggered at the beginning of the assay, and the ability of parasites to lyse a host cell monolayer was assessed after a weeklong incubation. Although the ability of the parental strain to generate plaques is undisturbed by the addition of IAA to the media (see Fig. S3), TgCep250L1-mAID-3HA parasites are unable to form plaques upon TgCep250L1’s knockdown, suggesting that TgCep250L1 is essential for survival (Fig. 1D).

10.1128/mbio.01859-22.3 FIG S3 IAA addition to the growth media has no effect on growth of the parental strain. The upper panel shows a plaque assay of the TgCEP250L1-iKD strain (RH DHXGPRT DKu80_HXGPRT^+^ *Os*Tir1_3FLAG_CAT^+^_Cep250L1-3HA-mAID) in presence or absence of IAA (shown in Fig. 1D). The lower panel shows a plaque assay performed under the same conditions on the parental strain (RH DHXGPRT DKu80_HXGPRT^+^ *Os*Tir1_3FLAG_CAT^+^). Although the total number of plaques is greater for the parental strain (likely due to a slightly larger number of plated parasites), note that the plaque size, when present, is comparable for both strains. Download FIG S3, PDF file, 0.07 MB. Copyright © 2022 Tomasina et al. 2022 Tomasina et al. https://creativecommons.org/licenses/by/4.0/ This content is distributed under the terms of the Creative Commons Attribution 4.0 International license.

To delve into the mechanism of death experienced by the TgCep250L1-mAID-3HA parasites, we analyzed *in vitro* growth. For this, we quantified the number of parasites per vacuole upon 24 h of protein knockdown. At that time point, the vast majority of mutant parasites presented two parasites per vacuole (80%). In contrast, only 40% of the control parasites exhibited two parasites per vacuole at the same time point. While ~55% of the control parasites exhibit four to eight parasites per vacuole, no vacuoles of eight parasites were observed in the IAA-treated parasites (Fig. 1E).

### TgCep250L1 knockdown causes nuclear segregation defects.

**FIG 2:**
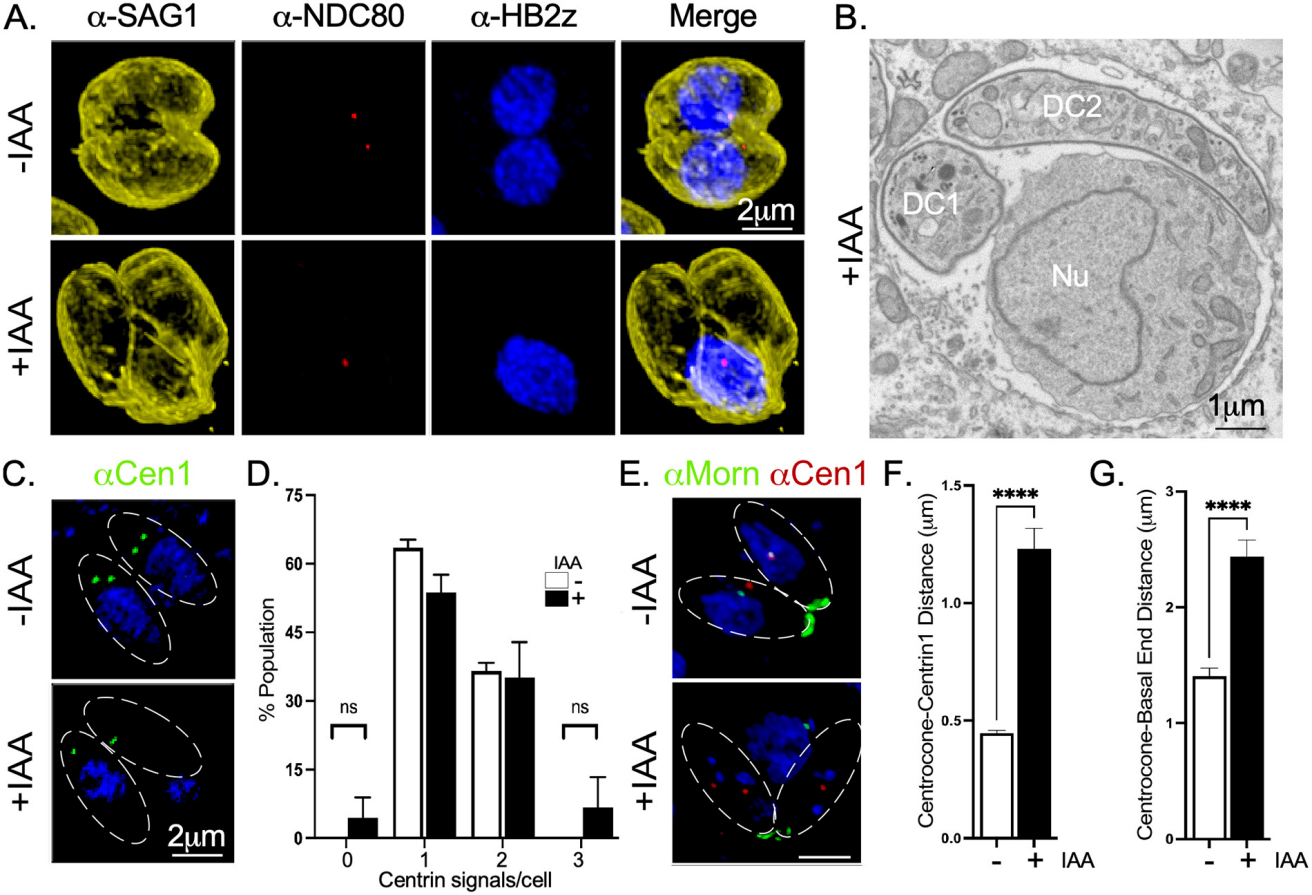
TgCep250L1 knockdown causes nuclear loss to the residual body. (A) Representative images of an IFA of TgCep250L1-mAID-HA parasites, stained with anti-Ndc80, anti-H2Bz, and anti-SAG1, markers of the kinetochore, nucleus, and plasma membrane, respectively, and treated as indicated are shown. The images shown correspond to maximum intensity projected z-stacks spanning the entire vacuole. Note that the plasma membrane marker SAG1 also labels the periphery of the residual body. (B) Electron micrograph of parasites treated with auxin for 24 h displaying an unsegregated nucleus within the residual body. Two individual cells (daughter cells 1 and 2 [DC1 and DC2], respectively) can be identified by the presence of the electron-dense inner membrane complex. A large nucleus (Nu) resides in the residual body, recognized at the ultrastructural level by its lack of inner membrane complex. (C) Outer core position and number are unaffected by absence of TgCep250L1. Representative IFA using anti-Centrin1 marks the position of the outer core of the centrosome (green) in parasites treated as indicated. The approximate parasite position is shown as dotted lines on the merge panel. Maximum intensity projected z-stacks, spanning the entire vacuoles, are shown. (D) Quantification of outer cores per cell. The number of centrin dots per cell was determined for parasites treated as indicated by IFA using anti-Centrin1 as a proxy of the outer core and DAPI. The data shown are the averages of three independent experiments. A minimum of 50 vacuoles were quantified per replicate. Data plotted are the average of three independent experiments. Error bars represent the standard deviations of the mean (n.s.; *P* ≥ 0.05). (E) Representative IFA results for parasites labeled and treated as indicated. Note that anti-MORN1 labels the base of the centrocone, an elaboration of the nuclear envelope that houses the mitotic spindle during cell division, and the basal end of the parasite. The linear distance between the outer core and either the centrocone (F) or the basal end of the cell (G) (labeled with anti-Centrin1 and anti-MORN1, respectively, as shown in panel E) was determined in parasites treated as indicated. The data shown are the averages of three independent experiments. A minimum of 50 vacuoles were quantified per replicate. Data plotted are the averages of three independent experiments. Error bars represent the standard deviations of the mean (****, *P* < 0.0001).

To understand the underlying defects giving rise to the mutant’s growth arrest, we performed IFAs upon TgCep250L1’s knockdown. We used anti-IMC1 to label the inner membrane complex, a structure that scaffolds the emerging daughter and marks the mother cell’s pellicle (14); anti-TgH2Bz, to label a histone variant (as a nuclear DNA marker) (15); and DAPI (4′,6′-diamidino-2-phenylindole; for general DNA labeling). Although TgH2Bz specifically labels a histone bound to the nuclear genome, DAPI labels both the nuclear chromatin and the apicoplast, an organelle of endosymbiotic origin present in most apicomplexans which bears its own genome (16). By 24 h after TgCep250L1 knockdown, we observed cell division progression. Although 40% of the vacuoles appeared normal (i.e., each parasite contains a single nucleus), 60% of the vacuoles exhibited parasites displaying nuclear segregation defects (Fig. 1F and G). A third of these defective-looking vacuoles (approximately 20% of the total) exhibited an individual containing an enlarged nucleus and individuals with either a minimal fragment or no detectable nuclear content. The remaining vacuoles (approximately 40% of the total) contained parasites whereby the nucleus has not segregated into either one of the forming cells but instead remained excluded from the parasites’ pellicle (Fig. 1G). Unpacked nuclei were observed by IFA, by labeling the plasma membrane with the marker TgSag1 (Fig. 2A). Observation of the latter by transmission electron microscopy (TEM) showed nuclei within a structure known as the residual body (Fig. 2B). Note that the residual body can be clearly distinguished from the daughter cells, since the former is only delimited by mother-cell derived plasma membrane (labeled in IFAs by TgSag1), while the latter displays the inner membrane complex (labeled in IFAs by TgIMC1), visualized by TEM as an electron-dense outline underlying the plasma membrane (17).

Nuclear segregation and daughter cell scaffold formation are physically linked to the centrosome (8, 18, 19). Cells in interphase display a single centrosome which is duplicated upon S-phase entry and then segregated during mitosis/cytokinesis. We assessed whether the nuclear segregation defect observed upon knockdown of TgCep250L1 could be linked to defects in the segregation of the centrosome itself. The outer core of the centrosome can be approximated by labeling the centriolar centrin with an anti-Centrin1 antibody (see Fig. S4). We labeled parasites with anti-Centrin1 and DAPI and quantified the number of Centrin1 dots per cell. We observed the expected distribution of either one or two centrin dots per cell, in both treated and untreated parasites (Fig. 2C and D; see also Fig. S5). We seldom detected parasites displaying either none or up to three centrin signals upon TgCep250L1’s knockdown; specifically, 7 of 150 cells displayed no centrin signal (4.6%), and 10/150 (6%) cell displayed three centrin dots. These exceptions, however, do not significantly alter the overall distribution. These analyses reveal that the absence of TgCep250L1 does not seem to significantly impact either the segregation or the duplication of the outer core the centrosome (Fig. 2C and D).

10.1128/mbio.01859-22.4 FIG S4 Outer and Inner core relative position observed by UExM. Intracellular dividing parasites were subjected to ultrastructure expansion (UEx) and stained with antibodies as indicated. Cell division is evidenced by the duplicated centrosomes in each nucleus. Note that a-H2Bz labels the nucleus. In expanded parasites, anti-TgCentrin1 staining (green) allows the resolution of both centrioles of the outer core in most centrosomes. The inner core (TgCep250L1, labeled with anti-HA in red) remains as a single dot distanced from the centrioles. Download FIG S4, PDF file, 0.10 MB. Copyright © 2022 Tomasina et al. 2022 Tomasina et al. https://creativecommons.org/licenses/by/4.0/ This content is distributed under the terms of the Creative Commons Attribution 4.0 International license.

10.1128/mbio.01859-22.5 FIG S5 TgCEP250L1 localization along the cell cycle. Intracellular parasites were subjected to UEx and stained with antibodies as indicated. Note that acetylated tubulin is present, and therefore labeled, by the specific antiserum in multiple structures, including the scaffold microtubules of both the mother and the forming daughter cells, the centrioles, and the mitotic spindle. Download FIG S5, PDF file, 0.2 MB. Copyright © 2022 Tomasina et al. 2022 Tomasina et al. https://creativecommons.org/licenses/by/4.0/ This content is distributed under the terms of the Creative Commons Attribution 4.0 International license.

TgMORN1 marks the base of the centrocone, a structure of the nuclear envelope known to house the mitotic spindle during mitosis, and the basal end of the parasite. Nuclear TgMORN1 is physically adjacent to the position of the centrosome (20) (Fig. 2E). To gain quantitative insight into the separation between the outer core of the centrosome and the nucleus upon TgCep250L1 knockdown, we analyzed the relative distance of TgCentrin1 with respect to that of nuclear TgMORN1. On average, TgCentrin1 and TgMORN1 are 447 nm apart (±103 nm; 150 nuclei) in untreated parasites. Upon IAA addition, the average distance approximately triples (1.23 ± 0.52 μm; 150 nuclei) (Fig. 2F). In untreated parasites, the average distance between basal end TgMORN1 and nuclear TgMORN1 is ~1.5 μm; this distance increases on average by 170% upon TgCep250L1 knockdown (2.4 ± 0.73 μm) (Fig. 2G). Taken together, these data strongly suggest that the mechanism underlying the cell division defect is caused by the disconnect of the outer core of the centrosome and cell pellicle from the nucleus.

### TgCep250L1 knockdown does not affect segregation of other organelles.

**FIG 3:**
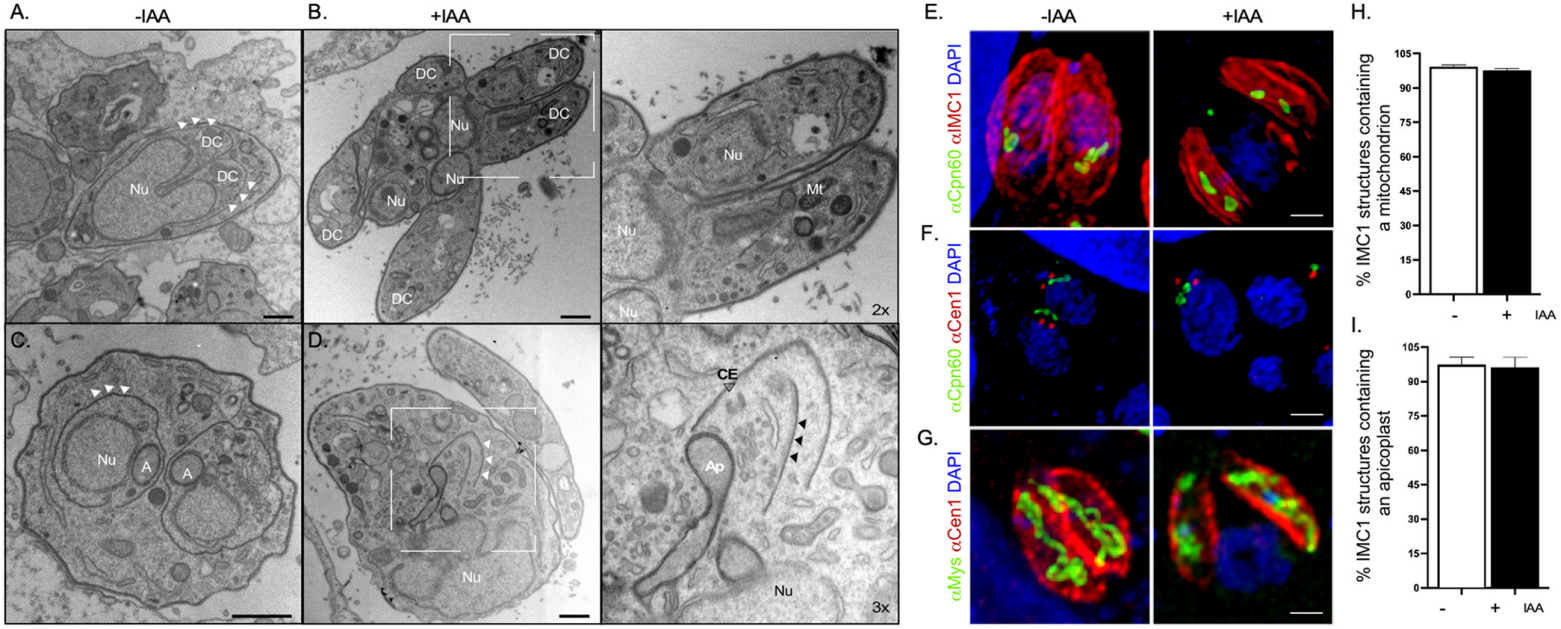
Lack of TgCep250L1 does not preclude segregation of the apicoplast or mitochondria. (A) Electron micrograph of a parasite dividing T. gondii cell (longitudinal view). The image shows the assemblage of two daughter cells scaffolds within an untreated mother cell displaying a bilobed nucleus (Nu) in the process of segregating to each daughter cell (DC). White arrowheads indicate the daughter cell’s inner membrane complex. (B) Ultrastructure of T. gondii parasites upon 24 h of TgCep250L1’s knockdown reveals proper mitochondrion segregation. Note that the T. gondii mitochondrion (M) is detectable within the forming daughter cell. However, several nuclei (Nu) remain excluded from the individual daughter cells. The inset shows an enlarged view of two of the daughter cells, wherein a mitochondrion profile (M) can be appreciated. (C) Electron micrograph of untreated T. gondii parasite dividing by endodyogeny (transversal view). Two daughter cells are assembled within a mother cell. A nucleus (Nu) is packed into each daughter cell. An apicoplast (A) that divides by association with the centrosome and hence segregates physically adjacent to the nucleus (Nu) and the centrocone (Cc) is shown. White arrowheads indicate the inner membrane complex of the daughter cells. (D) Ultrastructure of T. gondii parasites upon 24 h of TgCep250L1 knockdown reveals proper apicoplast segregation. The inner membrane complex of an assembling daughter cell is shown (white arrowheads). The apicoplast (A), a centriole (CE, empty arrowhead, inset), and the striated fiber (8) (black arrowheads, inset) are positioned as expected and are associated with the daughter cell scaffold. Note that nucleus (Nu), however, remains physically distant from the site of daughter cell assembly. All scale bars represent 500 nm. The zoom factor from the original image is indicated for each inset. (E to G) Projected z-stack of IFAs of parasites labeled and treated as indicated. α-Cpn60 was used as a marker of the apicoplast (E and F), while α-Mys marks the mitochondrion (G). Scale bars represent 1 μm. (H and I) Quantification of the number of cell scaffolds (as labeled with α-IMC1, as shown in panels E and G) containing either a mitochondrion (H) or an apicoplast (I). A minimum of 50 vacuoles were quantified per replicate. The data plotted are the averages of three independent experiments. Error bars represent the standard deviations of the mean. Note that there are no significant differences between treated and untreated parasites for the segregation of either the apicoplast or the mitochondrion.

During cell division, many of the preexistent mother cell organelles are duplicated and segregated into daughter cells. These include not only the centrosome and the nucleus but also both the apicoplast and the mitochondria, among others (19, 21). In particular, the apicoplast is segregated by association with the centrosome. To understand the extent to which the lack of TgCep250L1 affects general organelle segregation, we further investigated the ultrastructure of TgCep250L1-mAID-3HA parasites upon TgCep250L1 knockdown by TEM (Fig. 3A to D). In the IAA- treated population, we observe vacuoles containing daughter cell scaffolds that showcase both mitochondria and apicoplast (Fig. 3B and D, insets, labeled “Mit” and “Ap,” respectively). Consistent with our quantifications by IFA, we found dividing parasites showcase centrioles (housed within the outer core) within emerging daughters (Fig. 3D, inset, “Ce”), reinforcing the notion that the outer core is segregated correctly. In these vacuoles, we observed that nuclei are positioned away from the site of daughter cell assembly (Fig. 3B and D, inset, “Nu”). Of note, multiple centrioles within daughter cell scaffolds were never observed by TEM.

We further performed IFAs with either anti-IMC1 antibodies (to label the daughter cell scaffold) or anti-Centrin1, and anti-TgCpn60 to label the apicoplast (Fig. 3E and F). We observed that the organelle associates with the outer core, and its segregation is unaffected by the absence of TgCep250L1 (Fig. 3E, F, and H; see also Fig. S6). Likewise, labeling with anti-IMC1 and anti-Mys (a mitochondrial marker), revealed that mitochondria are properly segregated (Fig. 3G and I). Together, these data suggest that the nuclear segregation defect observed upon TgCep250L1 knockdown is not related to a general defect in cell division or to a defect in partitioning of the outer core of the centrosome between emerging cells but is instead specific to nuclear segregation.

10.1128/mbio.01859-22.6 FIG S6 TgCEP250L1 knockdown does not affect outer core duplication or segregation, nor does it affect the segregation of its associated organelles. Parasites subjected to IAA treatment were expanded, labeled with the indicated antibodies, and observed under confocal fluorescence microscopy. 1 and 2 refer to the pair of centrioles for each parasite. “Apico DNA” marks the apicoplast genome, which is labeled, together with the nuclear chromatin, by DAPI. Download FIG S6, PDF file, 0.09 MB. Copyright © 2022 Tomasina et al. 2022 Tomasina et al. https://creativecommons.org/licenses/by/4.0/ This content is distributed under the terms of the Creative Commons Attribution 4.0 International license.

### TgCep250L1 localizes at the site of the mitotic spindle assembly during mitosis.

To gain insight into the molecular mechanism underlying the nuclear missegregation phenotype of the TgCep250L1 knockdown cells, we set out to finely map the localization of TgCep250L1 along the cell cycle, with respect to structures known to participate of nuclear segregation. The centrosome of T. gondii is at the limit of optical microscopy resolution (~200 nm in size). Although much has been deciphered about the ultrastructure of the organelle by TEM, molecular insight into relative position of different elements (such as, for example, the inner and outer cores) has been incremental due to the use of super-resolution fluorescence microscopy technologies, such as structured illumination (9).

**FIG 4:**
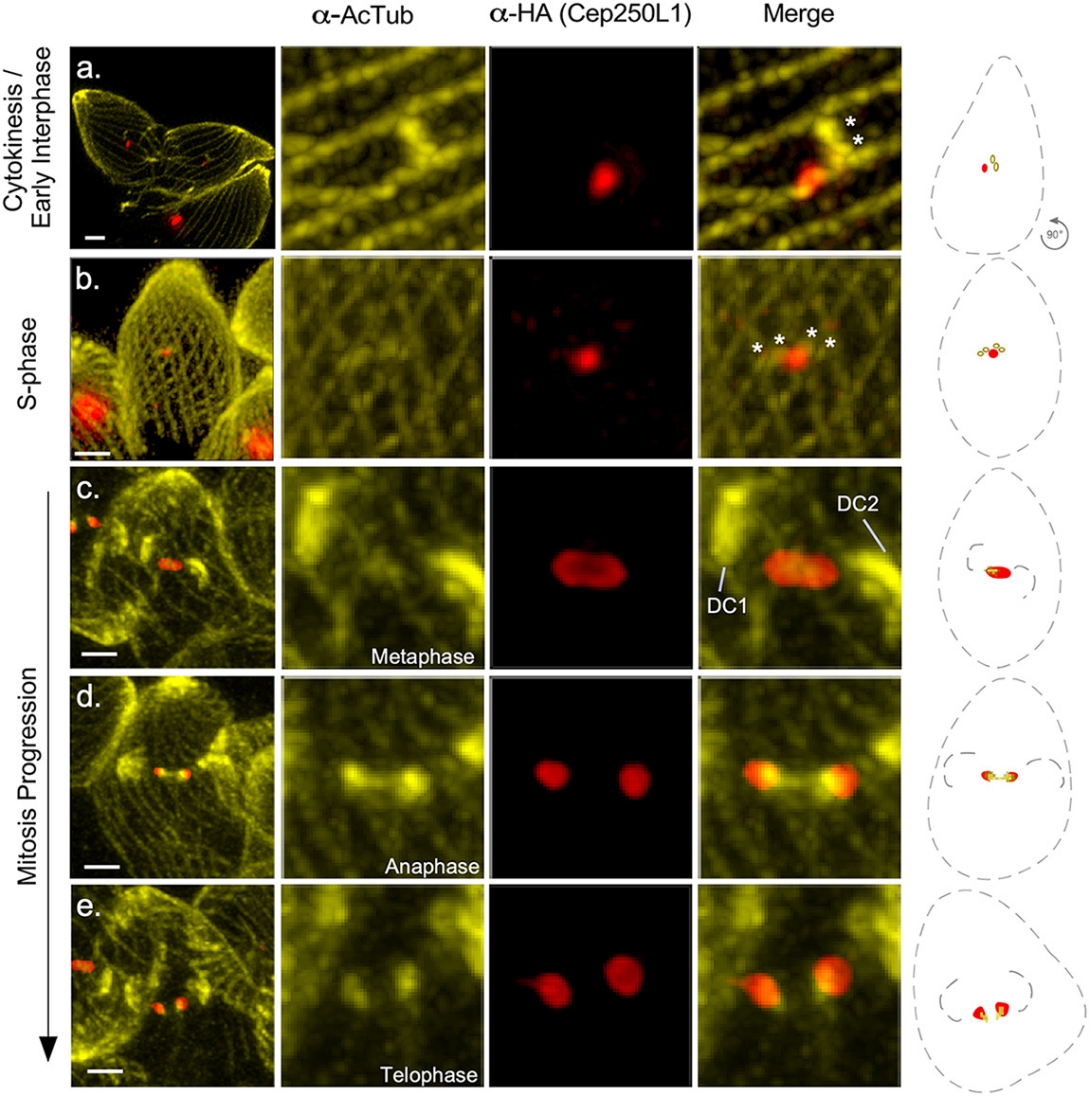
TgCep250L1 localization along the cell cycle. Parasites were subjected to ultrastructure expansion (UEx) and stained with antibodies as indicated. Note that acetylated tubulin is present, and therefore labeled, by the specific antiserum in multiple structures, including the scaffold microtubules of both the mother and the forming daughter cells, the centrioles, and the mitotic spindle. Asterisk indicate the position of individual centrioles. Daughter cells are labeled “DC1” and “DC2” All images shown are maximum-intensity projected z-stacks spanning the entire vacuole. The scale bar represents 1 μm in all cases. Schematic representations of the main structures labeled are shown. TgCep250L1 possition is shown in red (α-HA signal), the mother cell and daughter cell scaffolds are outlined by dotted lines, and the Ac-tubulin (acetylated tubulin) of the centrioles (a and b) or of the spindle (c to e) are shown in yellow.

A cost-effective alternative to the latter is the combined use of isotropic sample expansion and classical confocal fluorescence microscopy. Ultrastructure expansion microscopy (UExM) has been recently incorporated for routine use in T. gondii (22). We found that this technique allowed us to expand parasites, on average, ~3.5-fold. We labeled expanded parasites with an anti-acetylated tubulin antibody that labels the mitotic spindle, the centrioles, and the cortical microtubules (Fig. 4; see also Fig. S4 and S6). The increase in resolution allowed us to clearly visualize that, as reported previously, TgCep250L1 does not colocalize with the centrioles (see Fig. S4). We observed that in newly formed daughter cells (early interphase, defined by the presence of two centrioles) TgCep250L1 localizes adjacent to the centrioles (Fig. 4, merge panel a, “asterisks”). Upon entry into S phase, defined by centriole duplication, TgCep250L1 consistently localizes in between the duplicated pair (Fig. 4, merge panel b, “asterisks”) (21, 23). Shortly after duplication, the two pairs of centrioles move away from each other, and daughter cell scaffolds become apparent (7, 8). Samples fixed at this stage systematically display an oval-like shape localization for TgCep250L1 bridging the centrioles’ localization, and colocalizing with acetyl-tubulin positive staining (Fig. 4, subpanel c; daughter cells are marked as “DC1” and “DC2” [Fig. 6B, –IAA panel]; see also Fig. S5, “Early Cell Division” panel). Later in mitosis TgCep250L1 localizes to two distinct dots, each localizing at the tip of an anti-acetylated tubulin-labeled structure (Fig. 4, subpanels d and e; see also Fig. S5).

**FIG 5:**
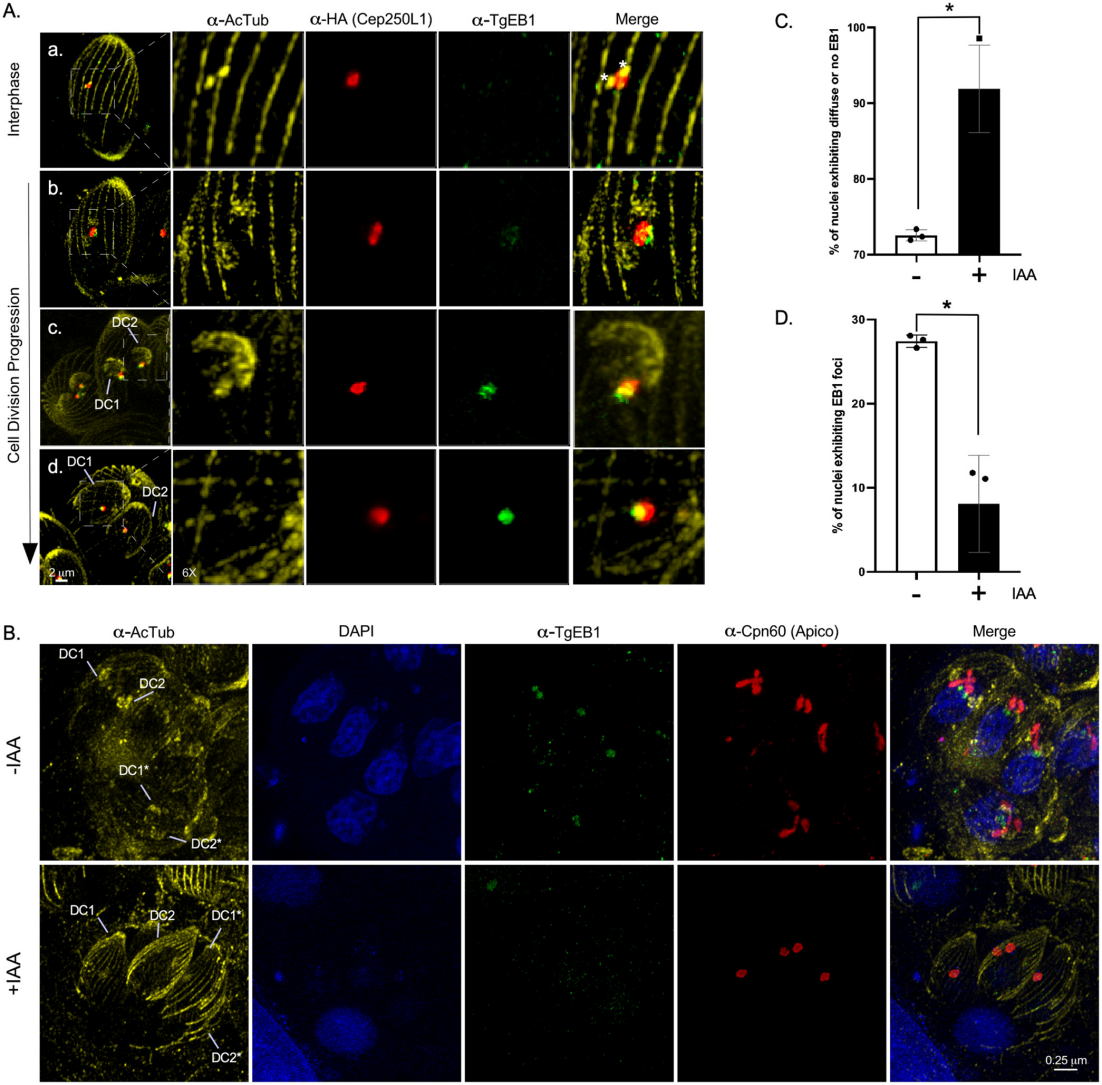
TgCep250L1 dynamics correlates with a microtubule binding protein of the mitotic spindle. (A) Parasites subjected to ultrastructure expansion were labeled with antibodies as indicated. Note that acetylated tubulin is present, and therefore labeled, by the specific antiserum in multiple structures, including the scaffold microtubules of both the mother and the forming daughter cells, the centrioles, and the mitotic spindle. An asterisk indicates the position of the individual centrioles. Daughter cells are labeled “DC1” and “DC2.” All images shown are maximum-intensity projected z-stacks spanning the entire vacuole. TgEB1 (green) is a mitotic spindle marker that displays a dynamic cell cycle-dependent pattern of localization (25). Note that in subpanel a, centrioles (marked with asterisks) can be individually resolved. At this stage, while TgCep250L1 is detectable, TgEB1 is not. Accumulates of ac-tub (acetylated tubulin), at the poles of the TgCep250L1 location, are perceptible in subpanel b. TgEB1 is detectable at this stage. Daughter cells (DC1 and DC2) are clearly identifiable in subpanel c. Larger daughter cell scaffolds (DC1 and DC2) are observable in subpanel d. At the latter two stages, TgCCep250L1 colocalizes with foci of TgEB1. (B) TgCep250L1-mAID parasites treated as indicated were subjected to ultrastructure expansion and labeled with antibodies as indicated. Note that the apicoplast marker α-Cpn60 was used as a proxy for the position of the centrosome. Note that when daughter cells (DCs) are barely detectable in untreated parasites, TgEB1 can be clearly seen concentrated at the periphery of the nucleus, in apposition to the apicoplast. In IAA-treated parasites, despite displaying larger and apparently well-formed daughter cell scaffolds and segregated apicoplast, no TgEB1 is detectable. (C and D) Quantification of the number of nuclei (as labeled with DAPI, as shown in panel B) displaying either a focus of TgEB1 (D) or nondetectable TgEB1 (C). A minimum of 50 vacuoles were quantified per replicate. The data plotted are the averages of three independent experiments. Error bars represent the standard deviations of the mean (*, *P* < 0.05).

The mitotic spindle is a key structure of the molecular machinery controlling chromosome segregation. The spindle is generally made up of dynamic microtubules that are decorated by microtubule binding proteins fulfilling different roles to ensure proper DNA segregation. Microtubule binding proteins of the EB1 family directly bind and stabilize microtubules in species ranging from yeast to human. In T. gondii, spindle microtubules are only polymerized during cell division and are absent during interphase (24). TgEB1 is a well-conserved member of the EB1 protein family that displays a dynamic localization, residing in the nucleoplasm outside of division, being virtually undetectable, but moving along with the spindle as it forms early in mitosis (see Fig. S7, –IAA panels) (25). In dividing parasites TgEB1 markedly localizes in between the centrioles, where the mitotic spindle resides (see Fig. S7, –IAA lower panel). Likewise, EB1 foci can be observed adjacent to the apicoplast during division, as the latter divides by association with the centrosome (Fig. 5B). Using UExM, we visualized the relative positions of TgCep250L1, TgEB1, and the spindle tubulin labeled with anti-acetylated tubulin (Fig. 5A). We observed that TgCep250L1 localizes in between duplicated centriole pairs prior to the time when TgEB1 is detectable at the spindle (Fig. 5Aa, centrioles are marked by asterisks). The time at which TgEB1 becomes appreciably detectable coincides with the “oval” staining pattern adopted by TgCep250L1 (Fig. 5Ab; see also Fig. S4). When TgCep250L1 localizes to the proximal tip of the tubulin structure labeled by anti-acetyl tubulin antibodies, TgEB1 localizes immediately beneath it (Fig. 5Ac; see also Fig. S5).

10.1128/mbio.01859-22.7 FIG S7 Knockdown of TgCep250L1 precludes TgEB1 translocation to the spindle. TgCep250L1-mAID-HA parasites were stained with antibodies and treated as indicated. In the presence of TgCep250L1, untreated parasites exhibit either largely undetectable TgEB1 outside of division (upper panel) or readily detectable TgEB1 in between the duplicated centrosomes (red) during division (lower panel). The latter corresponds with the expected position of the mitotic spindle. In treated parasites, where the TgCep250L1 is knocked down, TgEB1 remains in the nucleoplasm when detectable (both panels) and does not relocalize even in the presence of multiple centrosomes, which is indicative of cell division (lower panel). Download FIG S7, PDF file, 0.1 MB. Copyright © 2022 Tomasina et al. 2022 Tomasina et al. https://creativecommons.org/licenses/by/4.0/ This content is distributed under the terms of the Creative Commons Attribution 4.0 International license.

Using the apicoplast as proxy for the centrosome position (having previously established that the apicoplast and outer core segregate properly), we sought to determine whether TgEB1’s localization and/or dynamics are impacted upon TgCep250L1’s knockdown. We observed that in dividing parasites, displaying the mutant phenotype (i.e., fragmented or unsegregated nuclei), TgEB1 is undetectable (Fig. 5B, +IAA panel). We seldomly observe cumulates of TgEB1 at the nucleus, however, we never detect it at its expected position adjacent to TgCentrin1 (see Fig. S7, +IAA panels). To quantitate the extent of this defect, we quantified the presence of nuclei exhibiting diffuse or undetectable TgEB1, both in untreated and treated parasites (Fig. 5C). TgEB1 is undetectable in roughly 75% of untreated parasites (–IAA); this is expected, since about 75% of an asynchronously growing population will be in interphase at a given time. In contrast, >90% of parasites exhibit diffuse or undetectable TgEB1 upon TgCep250L1 knockdown. Conversely, TgEB1 foci could be detected (albeit not necessarily adjacent to the centrosome) in <10% of TgCep250L1 knockdowns, whereas >25% nuclei display TgEB1 foci in untreated parasites (Fig. 5D; see also Fig. S7 and S8).

10.1128/mbio.01859-22.8 FIG S8 TgCEP250L1 knockdown causes severe nuclear segregation defects. (A) Indirect immunofluorescence (IFA) of TgCEP250L1-mAID-3HA parasites labeled and treated as indicated. Note that the depletion of TgCep250L1 causes nuclear segregation defects, including the incorrect segregation of the nuclear content (marked by the histone H2Bz) and the kinetochores (marked by Ndc80) segregate. Defective Ndc80 segregation coincides with the lack of detection of the mitotic spindle marker (TgEB1). In contrast, daughter cells are fully formed. Download FIG S8, PDF file, 0.08 MB. Copyright © 2022 Tomasina et al. 2022 Tomasina et al. https://creativecommons.org/licenses/by/4.0/ This content is distributed under the terms of the Creative Commons Attribution 4.0 International license.

### The inner core resides with the chromatin-free region of the nucleus termed the centrocone.

**FIG 6:**
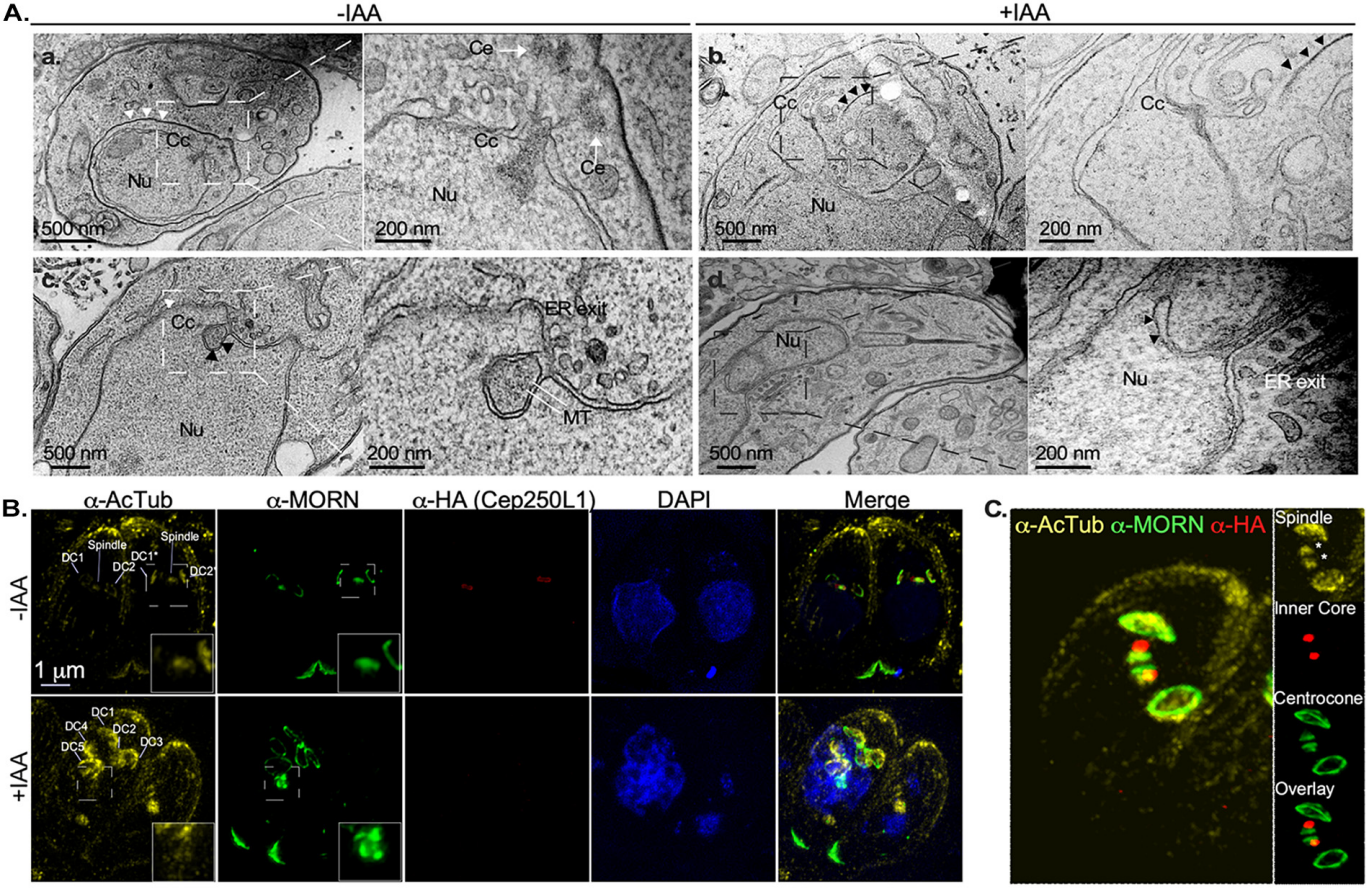
TgCep250L1 knockdown causes mitotic spindle assembly defects. (A) TgCep250L1 knockdown alters the centrocone structure The centrocone houses the mitotic spindle during cell division. A transmission electron micrograph of untreated TgCep250L1-mAID-3HA parasites (transversal view, subpanel a) shows the centrioles (Ce), centrocone (Cc), and segregating nucleus (Nu). Note that the centrocone houses electron dense material and that its position at the nuclear envelope is always adjacent to the endoplasmic reticulum (ER) exit site. White arrowheads indicate the inner membrane complex position of the assembling daughter cell. An additional electron micrograph of untreated TgCep250L1-mAID-3HA parasites (subpanel c) shows a view of the mitotic spindle microtubules (MT) housed within the centrocone (Cc), enveloped by nuclear envelope (black arrowheads), and is adjacent to the ER exit site. An electron micrograph (longitudinal view, subpanel b) of an IAA-treated parasite reveals a bilobed large nucleus, physically distant from the site of daughter cell assembly (black arrowheads indicate the forming daughter’s inner membrane complex). Note that adjacent to the ER exit site, an elaboration of the nuclear envelope, which is possibly reminiscent of the centrocone structure, can be appreciated. However, the structure is electron translucent. An additional electron micrograph of TgCep250L1-mAID-3HA parasites treated with IAA for 24 h parasites (subpanel d) shows a void invagination of the nuclear envelope adjacent to the ER exit site. Mitotic spindle microtubules are undetectable. (B) Parasites treated as indicated were subjected to ultrastructure expansion and labeled as indicated. Parasites undergoing division are shown. Daughter cells (DC) are indicated. The spindle microtubules are labeled by the anti-acetyl tubulin serum, albeit only visible in untreated parasites (α-AcTub panels, insets). Note that TgMORN1 localizes as expectedly in both treated and untreated parasites at the basal ends of mother and forming daughter cells. However, nuclear TgMORN1 accumulates aberrantly in +IAA-treated parasite’s nuclei (a-MORN panel, inset). Note that the images shown are maximum-intensity projected z-slices selected to clearly display the daughter cells’ scaffolds and spindles. The mother cell scaffold is not fully appreciable in these images. (C) Untreated parasites were subjected to ultrastructure expansion and labeled as indicated. A parasite undergoing division is shown. Insets show a detailed view of the relative localizations of the indicated markers. Spindles are marked by asterisks. Note that the conical shape of the centrocone can be resolved by combining UExM and α-MORN labeling.

We reckoned that the defect in TgEB1 recruitment during cell division could be underlay by defects in the formation or stability of the mitotic spindle. The mitotic spindle is known to assemble adjacent to the centrosome within a conical elaboration of the nuclear envelope, devoid of chromatin, adjacent to the ER exit site known as the centrocone (Fig. 6Aa and c) (20, 26, 27). Mitotic spindle microtubules can normally be detected within the centrocone in dividing parasites. The centrocone is visible as invaginations of the nuclear envelope (Fig. 6Ac, inset arrows “MT”). We detect nuclear envelope invaginations in IAA treated adjacent to the ER exit site (Fig. 6Ad). However, these are devoid of detectable microtubules. Conspicuously, we observe by TEM that IAA-treated parasites which failed to segregate their nuclei display protrusions of the nuclear envelope in the vicinity of the ER exit site (Fig. 6Ab). However, these protrusions only vaguely resemble the centrocone morphology during division, as they are devoid of electron dense material or detectable spindle microtubules (electron translucent structure labeled “CC” in inset of Fig. 6Ab).

The only known molecular marker for the centrocone is TgMORN1; it “decorates” first the opening at the nuclear envelope which persists outside of cell division, and later the conical structure that houses the mitotic spindle. As mentioned before, TgMORN1 also labels the parasite’s basal end from early in division, hence labeling coming daughter cells, as well as the mother’s base. We reckoned that the structural defects of the centrocone observed by TEM could be linked to the position of the inner core relative to the centrocone structure. We therefore explored TgMORN1’s localization upon TgCep250L1 knockdown. We observed by IFA that nuclei accumulate aberrant TgMORN1 signals specifically at the nucleus, while the protein localizes properly at the basal end of coming daughter cells displaying its characteristic ring-shape (Fig. 6B, +IAA panel; see also Fig. S9). We therefore wondered whether the inner core could in fact be contained within the centrocone itself. To explore this possibility we labeled expanded parasites with anti-TgMORN1, anti-acetyl tubulin to label the mitotic spindle, and anti-HA to label TgCep250L1. We observed that, indeed, TgCep250L1 is contained within the conical elaboration of the nuclear envelope formed during division, marked by TgMORN1, at a location which coincides with the tip of the site of spindle microtubule assembly (Fig. 6C). The overlay among the three markers persists throughout division (see Fig. S10A and B).

10.1128/mbio.01859-22.9 FIG S9 Defective TgMORN1 in mutant parasites. Intracellular IAA-treated parasites were subjected to ultrastructure expansion and labeled as indicated. Parasites undergoing division are shown. Daughter cells are discernable by the labeling of their scaffold by the anti-acetyl tubulin serum. Note that TgMORN1 localizes as expected at the basal ends of mother and forming daughter cells. However, nuclear TgMORN1 accumulates aberrantly in parasite’s nuclei. The image shown is a maximum-intensity projection of z-slices spanning the vacuoles shown. Download FIG S9, PDF file, 0.2 MB. Copyright © 2022 Tomasina et al. 2022 Tomasina et al. https://creativecommons.org/licenses/by/4.0/ This content is distributed under the terms of the Creative Commons Attribution 4.0 International license.

10.1128/mbio.01859-22.10 FIG S10 Relative disposition of elements involved in mitotic spindle assembly. (A) Untreated parasites were subjected to ultrastructure expansion and labeled as indicated. A parasite undergoing division is shown. (B) The two mitotic poles of dividing parasites (at an early stage of division) treated and labeled as in panel A were used the measure the linear disposition of the different elements involved in mitotic spindle assembly. (C) The relative distances among the inner core (TgCep250L1), the centrocone (TgMORN1), and the mitotic spindle (AcTub) were determined. Note that the signals of TgCep250L1 and TgMORN1 largely overlap in space. Separate staining of each marker can be found in the insets of Fig. 6C. Download FIG S10, PDF file, 0.1 MB. Copyright © 2022 Tomasina et al. 2022 Tomasina et al. https://creativecommons.org/licenses/by/4.0/ This content is distributed under the terms of the Creative Commons Attribution 4.0 International license.

### Absence of TgCep250L1 precludes proper DNA segregation.

We reckoned that if the loss of nuclear content displayed by the TgCep250L1 knockdown is linked to defects in mitotic spindle assembly, parasites should not only display “tossed out” nuclei but rather should suffer from DNA partitioning defects. To test this, we assayed two centromere-associated proteins whose distribution vary along with ploidy. We simultaneously assessed the parasite’s kinetochore marker TgNdc80 (18) and the centromeric histone TgCenH3 (28). In wild-type parasites, antibodies against either one of these proteins label a punctate structure at the nuclear periphery that arranges either as a single dot in nondividing parasites representing all centromeres/kinetochores bundled in one spot or as duplicated dots when chromatin and its associated structures (centromeres and kinetochores) have duplicated and began segregating.

**FIG 7:**
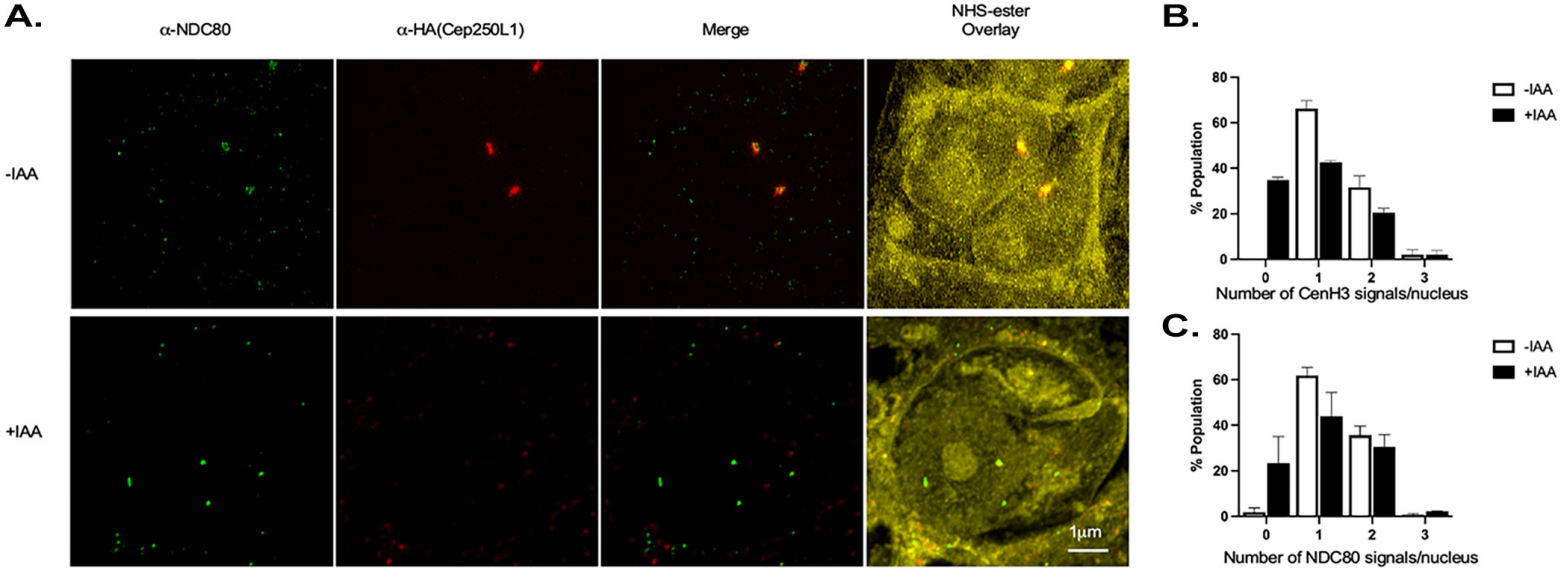
DNA segregation is aberrant upon TgCep350L1 knockdown. (A) Parasites treated as indicated were subjected to ultrastructure expansion and labeled as indicated. Note that the NHS-ester labels the pan-proteome, simultaneously labeling the nucleus, and the cell scaffold, among other structures. The lower panel (+IAA) displays multiple Ndc80 signals on an unsegregated/unpacked nucleus. Note that this is an infrequent finding, and it is shown to illustrate abnormal Ndc80 signaling. Images shown are maximum-intensity projections of z-stacks spanning the entire vacuole. (B) Quantification of the number of centromeres (labeled with anti-TgCenH3) per nucleus, showing that the untreated population exhibits the expected distribution of one or two centromeric foci. IAA-treated parasites, on the other hand, display one and two dots, but also accumulate DAPI-labeled DNA with no CenH3 signals. Parasites seldom exhibit an excessive number of centromeres per nucleus (>2) in the IAA-treated population, suggesting that segregation is affected but that polyploid nuclei are rare findings. (C) Quantification of the number of kinetochores (labeled with anti-Ndc80) per nucleus show that the untreated population exhibits the expected distribution of one or two kinetochore signals per nucleus. IAA-treated parasites, on the other hand, display one and two dots but also accumulate nuclei (DNA labeled with DAPI) with no Ndc80 signal. Parasites rarely exhibit an excessive number of kinetochores per nucleus (>2, as shown in lower part of panel A) in the IAA-treated population.

Quantification of the number of kinetochore/centromeres per nucleus shows that the untreated population exhibits the expected number of nuclei displaying 1 and 2 dots (Fig. 7). However, IAA-treated parasites showcase a significant decrease in the number of nuclei displaying one or two dots, and a concomitant increase in nuclei displaying 0, for both markers (Fig. 7). Rarely, nuclei displaying three or more dots are detected (Fig. 7). The significant accumulation of nuclei with no Ndc80/CenH3 signal, however, suggests either unequal DNA segregation, whereby some nuclei inherit no centromere/kinetochore, or fragmentation of the signal, rendering it below the limit of detection by fluorescence microscopy (Fig. 7A and B). Overall, these data support the notion that not only does the nucleus disconnect from the outer core, consequently disconnecting from the cell scaffold but also that proper DNA segregation to the poles of the undivided nucleus is impaired. The latter is consistent with defective assembly or maintenance of the mitotic spindle.

## DISCUSSION

TgCep250L1 is the only marker known to exclusively localize at the inner core of the T. gondii centrosome. In this study, we finely define its localization and described its function as a proxy for the role of the inner core. We observed that upon depletion of TgCep250L1 parasites exhibit nuclear segregation defects, while forming daughter cells containing other correctly segregated organelles. We further described the localization of TgCep250L1 as cell cycle, and particularly as spindle formation, progress. We found that TgCep250L1 displays a more complex localization pattern than previously appreciated, which particularly correlates with mitotic spindle formation during mitosis. Finally, we demonstrated that the spindle marker TgEB1 and acetyl-tubulin are absent upon TgCep250L1’s knockdown in dividing parasites, suggesting that either the formation or the stability of the mitotic spindle is affected. Finally, we show that as a consequence of the defect in spindle assembly, DNA segregation is aberrant in mutant parasites.

Animal centrosomes display a protein called c-NAP1 which participates in physically linking centrioles during interphase. Phosphorylation of c-NAP1 catalyzes the dissolution of the linkage between mother and daughter centrioles. Centriole separation in S-phase allows not only their duplication, forming a new centrosome, but it is also required for their separate migration to opposite poles of the cell. This process is catalyzed prior to mitosis by a NIMA (never in mitosis, gene A)-related kinase, called Nek1. The T. gondii genome encodes two homologs of c-NAP1, TgCep250 and TgCep250L1, both of which were initially described by Suvorova et al. (9). These were identified principally based on the presence of coiled-coiled domains (9). However, later *in silico* studies showed that both proteins are only distantly related to c-NAP1 (29). A conditional mutant of TgNek1 results in parasites displaying a single centrosome, suggesting that this kinase regulates centrosome splitting in T. gondii in an akin manner to that described for animal Nek1 (7, 11). However, it was experimentally proven that TgCEP250 is not a substrate of TgNek1 (11). On the other hand, TgCep250L1 is located at the inner core, distant from the outer core where centrioles reside, and TgNek1 is expected to exert its activity (9). The functional homolog of c-NAP1 and substrate of TgNek1 remain to be deciphered.

It was established in the early 60s by TEM that a mitotic spindle assembles adjacent to the centrioles of T. gondii (4, 30). Microtubules remain extranuclear, only penetrating the nuclear membrane at the base of a conical elaboration of the envelope, a structure coined more recently “the centrocone,” molecularly marked by the presence of TgMORN1 (31). Previous studies have addressed the dynamics of the mitotic spindle, showing that recruitment of tubulin to the centrocone happens at the end of interphase/G_1_, just before the onset of S phase, a stage marked by duplication of the centrioles (20, 21, 23, 25). The chromosomes, through their centromeres and kinetochore proteins, are associated with the centrocone throughout the cell cycle but are only reached by the microtubules of the spindle during mitosis (18, 24, 28). Although the ultrastructure of the mitotic spindle and its dynamics have been broadly described, the molecular pathways involved in the nucleation of the microtubule in T. gondii remain poorly understood.

In animal cells, the pericentriolar material (PCM) serves as a platform for protein complexes that regulate organelle trafficking, protein degradation and spindle assembly. This complex and dynamic matrix of proteins visibly envelopes the centrioles. A vast number of protein components of the PCM have been identified in species ranging from humans to flies. These include, but are not limited to, CEP152, CEP57, CPAP (SAS-4), CEP192, pericentrin, CDK5RAP2 (Cnn), and a number of microtubule binding/stabilizing proteins, together with regulatory kinases and phosphatases (including Polo-like kinase 1, Aurora A kinase, and PP2 homologs). Strikingly, many of the functionally most important PCM proteins seem to be absent from the T. gondii genome (9, 29, 32). On the other hand, γ -tubulin, a crucial protein participating in spindle microtubule nucleation through the nucleation of the γ-tubulin ring complex (γTuRC) in other eukaryotes, has been previously localized to the outer core of the centrosome (9, 33). It is noteworthy that microtubules of the cortical cytoskeleton of the daughter cell are nucleated at this location. Hence, the function of γ -tubulin and the uncharacterized γ-tubulin ring complex (γTuRC) could be circumscribed to nucleation of scaffold microtubules rather than the spindle.

Our results support the function of the inner core as a key element for organizing or stabilizing the mitotic spindle and further support the notion that an “in tandem” organized centrosome structure is the basis of compartmentalizing functions providing the physical and temporal coordination of cell cycle events in T. gondii. The modular nature of cell division in T. gondii (i.e., nuclear division and budding operate through seemingly independent regulatory networks) allows this parasite to divide using distinct modes, including endodyogeny and schizogony. In endodyogeny, nuclear mitosis is immediately followed by budding, while in schizogony budding only occurs following multiple rounds of nuclear mitosis. When failing, these modes of division result in the unusual generation of multiple zoites devoid of nuclear material. This is quite unusual in nature, whereby most cell division failures culminate in the unequal segregation of the genetic material between mother and daughter. The dual organization of the T. gondii centrosome has been repeatedly proposed to contribute to this cell division flexibility, whereby the outer core determines the number of daughter cells assembled while the inner core controls nuclear events, separately, overriding the classical “checkpoint” notions.

Our deep understanding of schizogonic cell division comes from studies of the *Plasmodium* genus. However, central structural differences exist between T. gondii and *Plasmodium* that raise questions about the conservation of certain regulatory principles between the schizogony in these species. One such difference is that T. gondii displays microtubule-based centrioles, whereas *Plasmodium* species do not. Plasmodium falciparum nuclear MTOCs is known as the centriolar plaque (CP). Until very recently, it was considered that the CP resided within the nuclear envelope, much like the budding yeast spindle pole body does. However, new advances in microscopy (including the combination of UExM with STED and CLEM) allowed Simon et al. to observe and describe an MTOC made of a bipartite structure; an extranuclear segment housing markers such as centrin, and an intranuclear segment housing tubulin (34). This study helped clarify that microtubule nucleation during schizogonic mitosis occurs at an intranuclear site devoid of chromatin rather than at the nuclear envelope itself. Concomitantly, Liffner and Absalon showed that the spindle undergoes dynamic changes which accompany the chromatin dynamics as mitosis progresses (35). Nondividing nuclei sustain a handful (~5) of bundled individual intranuclear microtubules that have been named the “hemi-spindle.” Upon DNA replication onset in S phase, the hemi-spindle retracts, the CP duplicates, and the mitotic spindle forms (35, 36).

Overall, these new molecular and structural insights suggest that while the nature of the nuclear MTOCs are different between species (i.e., centrioles versus no centrioles), the organization principle underlying positioning and activity of the nuclear MTOCs with respect to the nuclear envelope and chromatin are more similar than previously appreciated. Nonetheless, no homologs of TgCep250L1 are present in the *Plasmodium* genomes, suggesting that the molecular players differ.

Despite of the significant recent advances in our understanding of the centrosome structures in other *Apicomplexa*, the centrosome of T. gondii continues to be the most extensively studied and best understood within the phylum. The functional domains, the molecular players and regulatory enzymes, identified have increased significantly in recent years. Rather than a bipartite organelle, we now understand that the centrosome in T. gondii can be regarded as a continuum, delimited at its proximal end by the centrioles and at its basal end by the base of the centrocone, the kinetochores and the centromeres. In this context, TgCep530, was shown to localize in between the outer and inner cores, for the first time defining the “middle core” region. Conspicuously, inducible knockdown of TgCep530 renders parasites displaying enucleated parasites and mis-segregated nuclei. However, the TgCep530 mutant display additional outer core fragmentation (12), a phenotype we did not observe. This suggests that while the stability of the continuum may be required to coordinate karyokinesis with cytokinesis, middle core function may additionally be to regulate centriole/outer core stability. Consistent with the idea that cores are subject to differential regulation, a temperature-sensitive mutant of a mitogen-activated protein-related kinases (MAPKs) displays overduplication of Centrin1 but not of TgCep250L1 (9). Here, we show that lack of TgCep250L1 does not affect centriole duplication, suggesting that changes at the inner core are not directly transduced to the outer core. Consistently, depletion of TgArk-1, an aurora-related serine-threonine kinase which accumulates in the centrocone region, leads to underduplication of both the spindle pole and the inner core (37), with the unperturbed independent expansion of the outer core. The physical compartmentalization of proteins to distinct domains makes it so that defects at the middle core and inner core are rather circumscribed to the assembly or stability of the centrioles and the mitotic spindle, respectively.

Recently, a study describing the function of cyclin-dependent-related kinase 6 and its associated atypical cyclin 1 showed the complex to interact with the centromeric protein TgCenH3 and to be required for cell cycle progression past metaphase. Though it was shown that duplication of both the outer and inner core were unaffected, the assembly or stability of the mitotic spindle, however, was not directly explored in these mutants (38). Nonetheless, a conditional knockdown of MAPK2 displays concomitant underduplicated Centrin1 and TgCep250L1, suggesting that despite the modular regulation of the cores, an overall regulatory mechanism positively regulates the coordinated duplication of (possibly) all cores of the centrosome (39). It would be interesting to understand how the relative activities of these regulatory kinases (and the still to be identified counteracting phosphatases) interplay along the life stages of T. gondii whereby the parasite adjusts its cell division mode from endodyogeny to schizogony, modifying the relative temporal execution of each event of the cell cycle.

Future studies addressing the cell cycle-specific interactions of TgCep250L1 and their interplay with other elements of the multiple centrosomal cores and the regulatory enzymes of mitotic spindle assembly and the centromere/kinetochore function will shed light on the detailed mechanisms of action of this protein at the region of the nuclear periphery it inhabits and on the peculiar organization of the centrosome in T. gondii.

## MATERIALS AND METHODS

### Parasite culture.

T. gondii tachyzoites of the RHΔKu80 strain expressing the Tir1 receptor (13) were maintained in Vero cells grown in Dulbecco modified Eagle medium (Gibco, St. Louis, MO) and supplemented with 10% fetal bovine serum (Gibco), 4 mM l-glutamine (Gibco), and 200 U/mL of penicillin and 200 μg/mL of streptomycin (Gibco). Cultures were kept at 37°C and 5% CO_2_.

### Generation and preliminary characterization of the TgCep250L1 inducible knockdown strain.

TgCep250L1-mAID-3HA was generated in the RHΔku80 Tir1 strain background (40). Briefly, a PCR product of the mAID-3HA sequence bearing 35 bp of homology to either end of the TgCep250L1 gene stop codon was amplified using the primers 5′-GCACAGGAAGGGGAAAGTCGTCGCTTTCGGGGCGA-3′ and 5′-CTGTAGGCCGCATGTTTTCATTTTCCTCTTCACAC-3′. A plasmid coding for a guide RNA targeting the 3′ end of the TgCep250L1 gene and SpCas9 was generated by mutagenesis using the primers 5′-ACACTCTACGAACCTGTCCGCGGGTTTTAGAGCTAGAAATAGCAAGTTAA-3′ and 5′-AACTTGACATCCCCATTTAC-3′. Then, 50 μg of pSagCas9 vector and 10 μg of PCR product were transfected into 5.0 × 10^7^ parasites using a BTX 600 electroporator according to previously published protocols (24). The pSagCas9 vector (41) was kindly provided by David Sibley. Successful insertion of the 3HA-mAID sequence was monitored by PCR using the primers a (5′-GAGACTCAGAGCGCAAGACG-3′), a* (5′-CCCCGAAAGCGACGACTTTCCC-3′), and b (5’TTAGGCATAATCTGGAACATCG-3′), according to the schematic shown in Fig. S1A. Clonal cell lines were obtained by limiting dilution. Protein degradation was triggered by the addition of 0.5 mM indoleacetic acid (IAA; Sigma-Aldrich) to the growth medium.

### Plaque and intracellular growth assays.

For plaque assay, two hundred parasites of the TgCep250L1-mAID-3HA strain were inoculated on human foreskin fibroblast cells (HFFs; kindly provided by Sebastian Lourido) previously grown to confluence on six-well plates and kept for 7 days in presence or absence of 0.5 mM IAA. Wells were then fixed with methanol and stained with crystal violet for plaque visualization. Intracellular growth was determined by immunofluorescence assay (IFA; according to the protocol specified below) labeling parasites with the pellicle marker anti-IMC1 and DAPI (nuclear and apicoplast DNA marker). Assays were performed by infecting confluent HFF cells grown on 13-mm coverslips with 1,000 parasites. Parasites were allowed to invade and grow for 2 h prior to IAA addition to the media, when appropriate. Note that the medium was changed at the same time for parasites grown under control conditions (no IAA). Parasites were allowed to grow for an additional 24 h, prior to fixation and processing. Assays were performed in triplicate. Quantification was done by determining the number of parasites per vacuole in 35 randomly acquired fields (~100 vacuoles per experiment) using an Olympus epifluorescence microscope.

### Western blotting.

Total proteins were extracted from 1.0 × 10^8^ parasites grown on media supplemented with 0.5 mM IAA at different times or as indicated in the figure legends. Total proteins were extracted by resuspending the cell pellet in Laemmli buffer and boiling for 5 min. Protein samples were run on a 10% SDS-polyacrylamide gel and transferred onto a nitrocellulose membrane overnight. Primary and secondary antibody incubations were done in 5% milk–PBS with rabbit anti-HA at 1:500 (Cell Signaling, catalog no. 3724S) and anti-rabbit HRP at 1:10,000 (Bio-Rad, catalog no. 1721017). Images were obtained using an ImageQuant 800 Western blot imaging system (Amersham) with exposure for a total of 30 s.

### Optical microscopy.

IFAs were performed as previously reported (24). In short, HFFs were grown on coverslips and inoculated with parasites. Depending on the assay, intracellular parasites were fixed at different times using methanol for 5 min at −20°C or 4% formaldehyde for 20 min at room temperature. For primary antibodies, we used mouse anti-centrin at 1:1,000 (Cell Signaling, catalog no. 04-1624), rabbit anti-HA at 1:200 (Cell Signaling, catalog no. 3724S), mouse anti-IMC-1 (42), at 1:500 (kindly provided by Gary Ward, University of Vermont), guinea pig anti-TgEB1 (25) at 1:3,000 (kindly provided by Marc-Jan Gubbels, Boston College), rabbit anti TgH2Bz (15, 43) at 1:3,000 (kindly provided by Sergio Angel, INTECH-Chascomus), guinea pig anti-Ndc80 (18) at 1: 2,000 (kindly provided by Marc-Jan Gubbels, Boston College), rabbit anti-Cpn60 (44) at 1:3,000, rabbit anti-Mys at 1:2,000 (45), and mouse anti-acetylated tubulin at 1:1,000 (Sigma, catalog no. T7451). Goat anti-rabbit Alexa Fluor 405 (Invitrogen, catalog no. A-31556), goat anti-rat Alexa Fluor 488 (Invitrogen, catalog no. A-11006), goat anti-mouse Alexa Fluor 488. (Invitrogen, catalog no. A28175), goat anti-rabbit Alexa Fluor 488 (Invitrogen, catalog no. A-11008), goat anti-guinea pig Alexa Fluor 594 (Invitrogen, catalog no. A-11076), goat anti-rabbit Alexa Fluor 647 (Invitrogen, catalog no. A27040) and goat anti-mouse Alexa Fluor 647 (Invitrogen, catalog no. A-21235) were all used at a dilution of 1:2,000. Coverslips were mounted onto glycerol or fluoroshield, with DAPI, when appropriate.

Ultrastructure expansion microscopy (UExM) was performed as described previously (46) without modifications. The primary and secondary antibodies were used twice as concentrated than specified for IFA above for the UExM experiments.

All images were acquired using a Zeiss confocal LSM880 microscope using a Plan-Apochromat 63×/1.40 oil objective. All images were acquired and processed using the Zeiss ZEN blue edition v2.0 software. All images were deconvolved using Huygens Professional v19.10.0p2 64b (Scientific Volume Imaging, The Netherlands).

Quantifications were done on *z*-projected images spanning the entire vacuole, using the ImageJ measurements tool when appropriate. Results were plotted using GraphPad Prism v9.3.1. For all quantifications, three biological replicates were performed. A minimum of 50 nuclei or cells were quantitated for each condition, per experiment. Statistical analyses were carried out using GraphPad Prism v9.3.1 analytical tools. Unpaired two-tailed *t* tests with Welch’s corrections (parametric) were carried out to determine the statistical significance of the differences detected. Differences were considered significant if the *P* values were <0.05.

### Transmission electron microscopy.

TEM sample preparation was done according to previously published protocols (47). In short, intracellular parasites were fixed in 2.5% glutaraldehyde–0.1 M sodium phosphate buffer for 2 h at room temperature. The fixative solution was washed out three times with 0.1 M sodium phosphate buffer, and infected cells were postfixed with 1% OsO_4_. Dehydration was done sequentially incubating samples in 30, 50, 70, and 90% and pure acetone for 10 min each. Embedding was done using epoxy resin (PolyBed resin; Polysciences, Inc., Warrington, PA). Ultrathin sections were obtained, stained, and observed in a JEOL transmission electron microscope coupled to a digital camera.
