# Supplementary figures and images for: Separate To Operate: the Centriole-Free Inner Core of the Centrosome Regulates the Assembly of the Intranuclear Spindle in Toxoplasma gondii
Source: mBio. 2022 Sep 7;13(5):e01859-22. doi: 10.1128/mbio.01859-22 (PMC9600614; doi:10.1128/mbio.01859-22)

Supplementary Figure 1

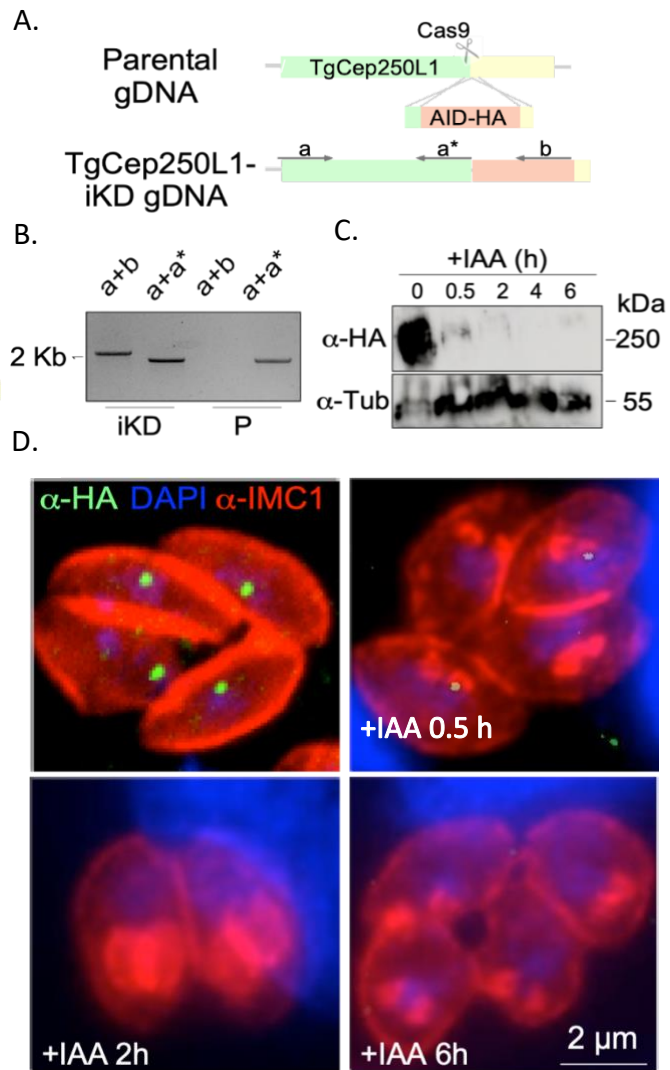

Supplement: FIG S1 [file mbio.01859-22-s0001.pdf]

Supplementary Figure 2

A.

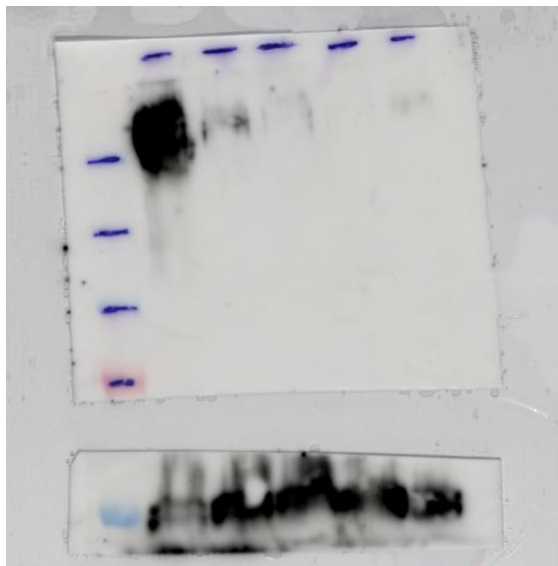

B.

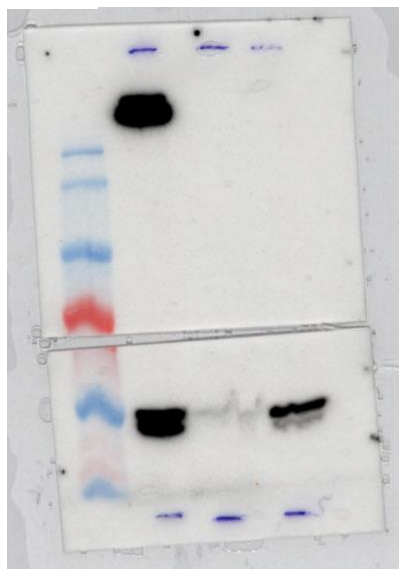

Supplement: FIG S2 [file mbio.01859-22-s0002.pdf]

Supplementary Figure 3

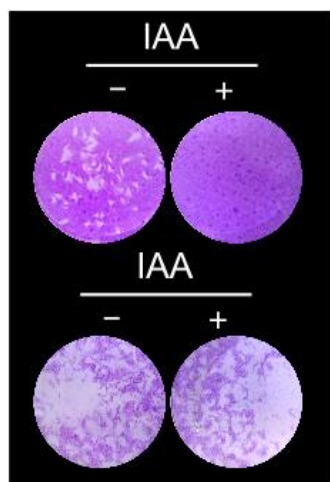

Supplement: FIG S3 [file mbio.01859-22-s0003.pdf]

Supplementary Figure 4

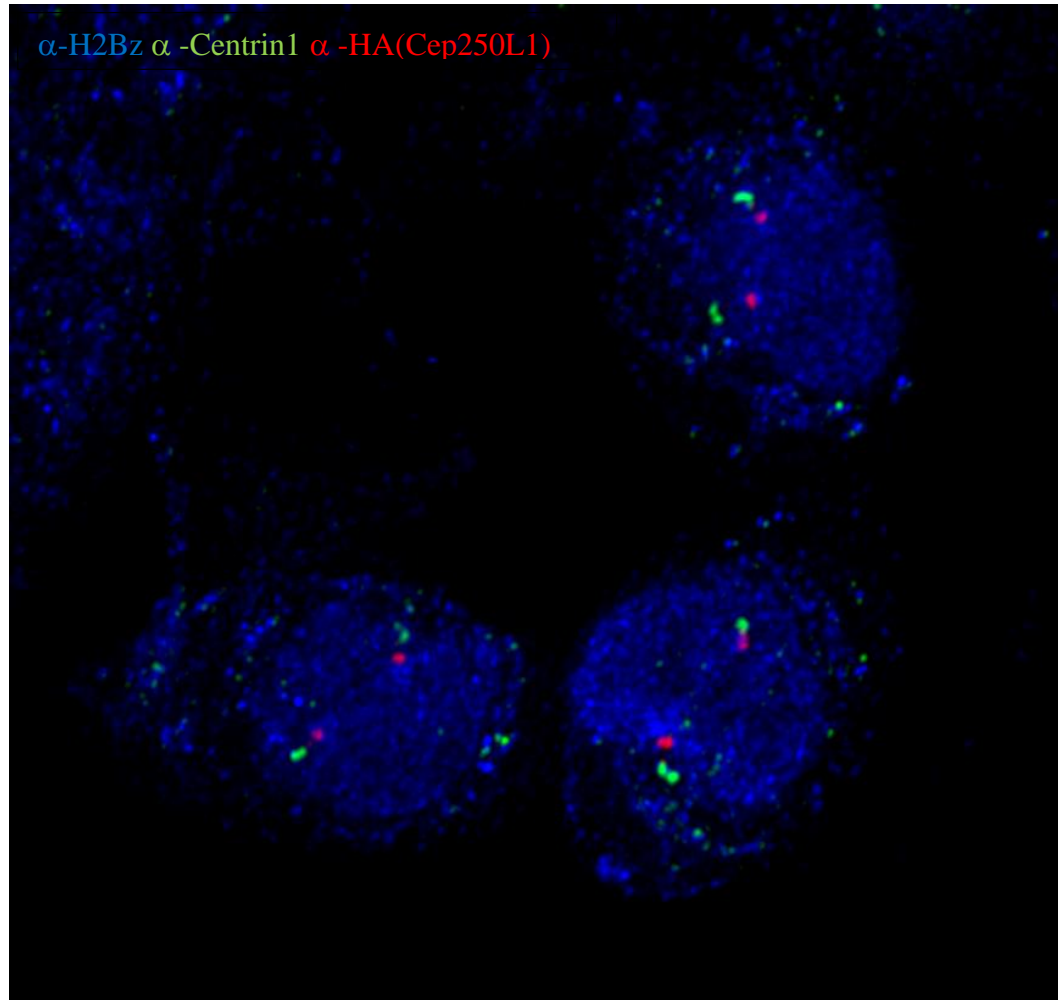

Supplement: FIG S4 [file mbio.01859-22-s0004.pdf]

Supplementary Figure 5

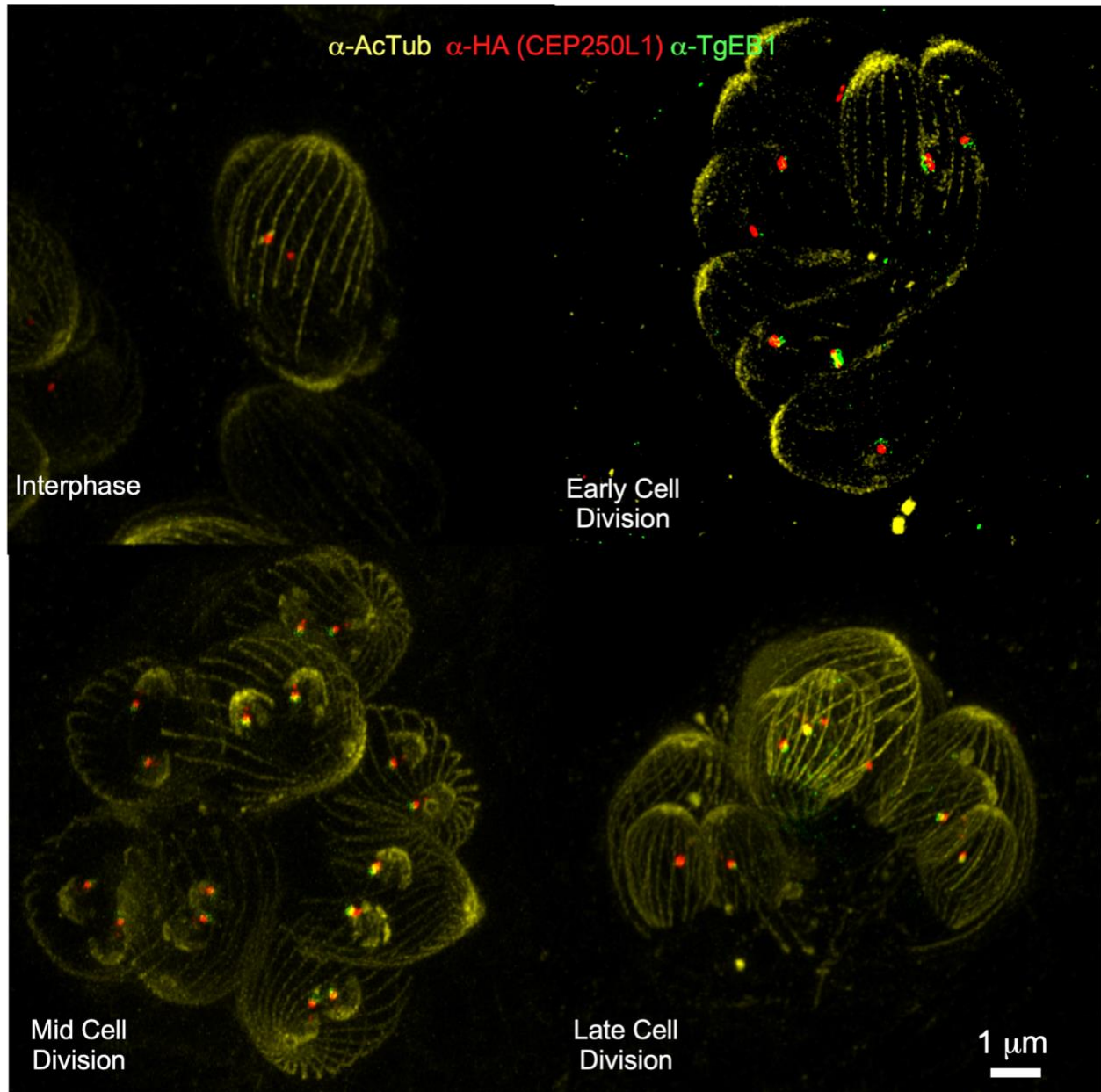

Supplement: FIG S5 [file mbio.01859-22-s0005.pdf]

Supplementary Figure 6

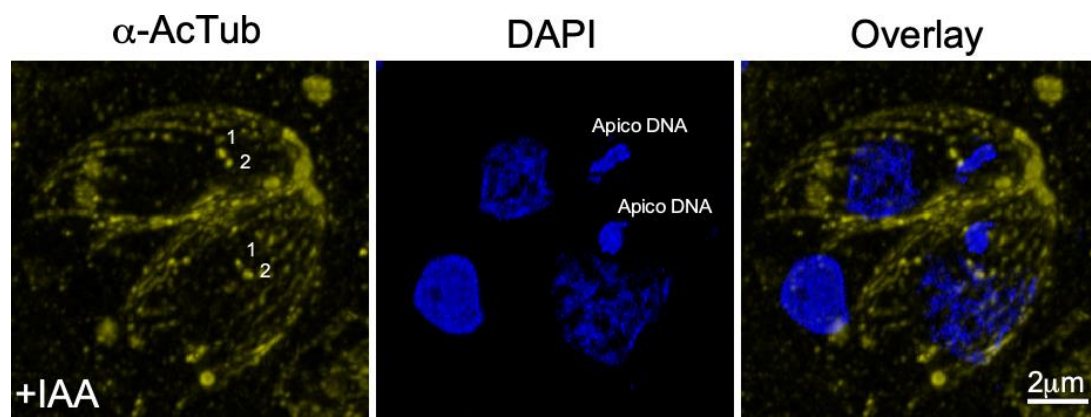

Supplement: FIG S6 [file mbio.01859-22-s0006.pdf]

Supplementary Figure 7

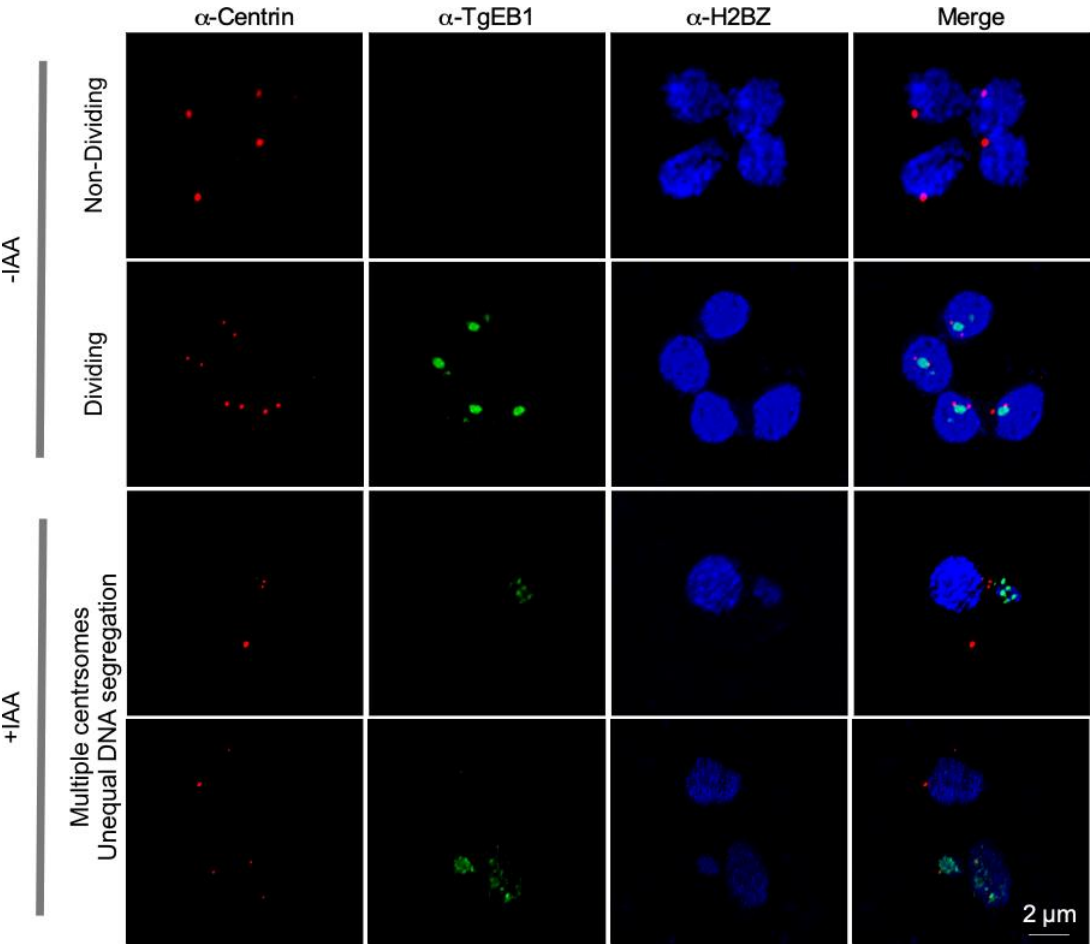

Supplement: FIG S7 [file mbio.01859-22-s0007.pdf]

Supplementary Figure 8

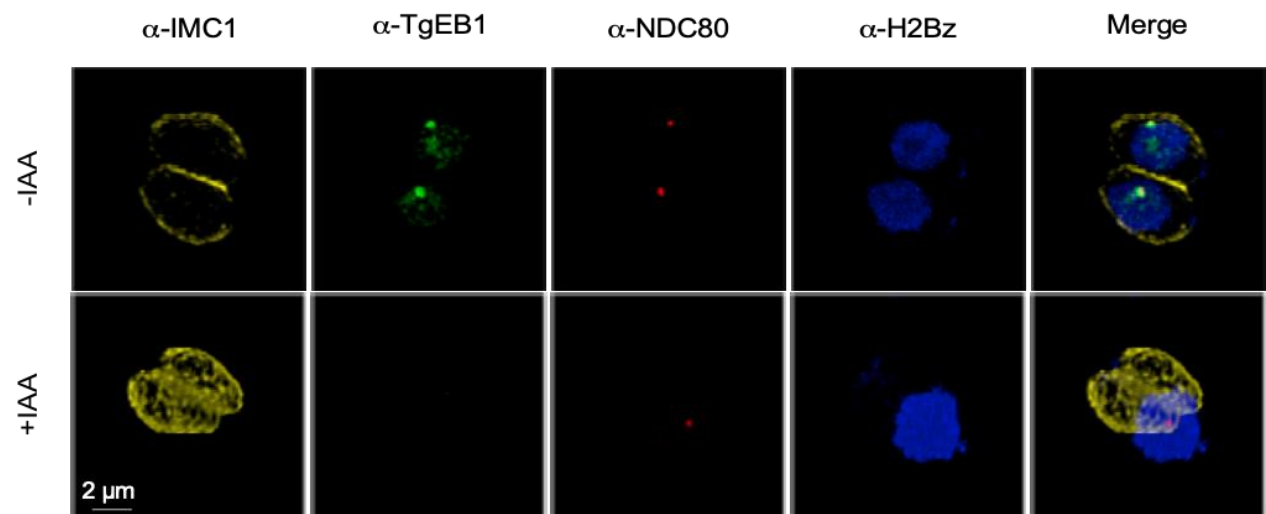

Supplement: FIG S8 [file mbio.01859-22-s0008.pdf]

Supplementary Figure 9

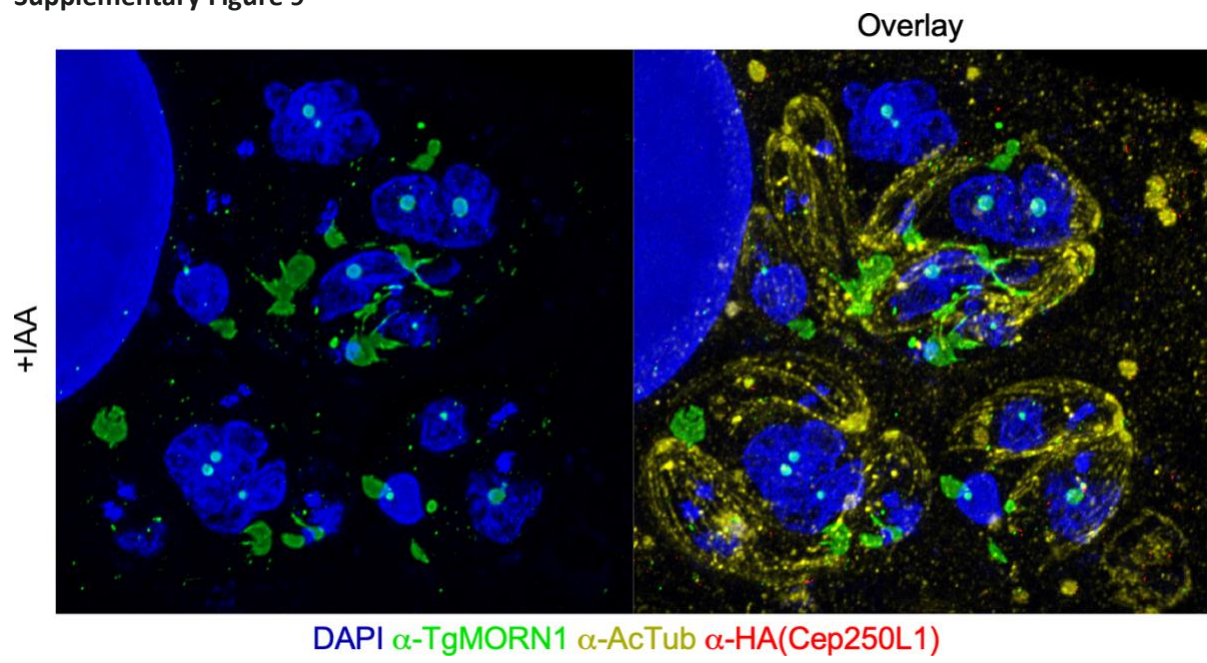

Supplement: FIG S9 [file mbio.01859-22-s0009.pdf]

Supplementary Figure 10

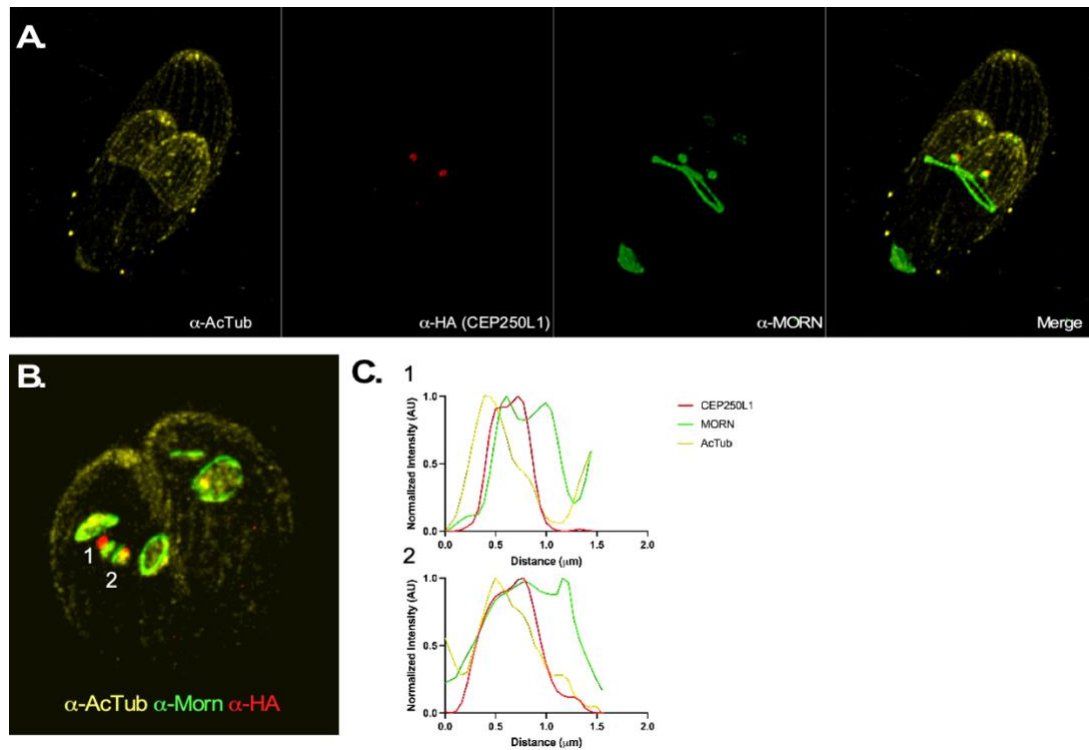

Supplement: FIG S10 [file mbio.01859-22-s0010.pdf]
